# Draft genome assembly of the Bengalese finch, *Lonchura striata domestica*, a model for motor skill variability and learning

**DOI:** 10.1093/gigascience/giy008

**Published:** 2018-02-15

**Authors:** Bradley M Colquitt, David G Mets, Michael S Brainard

**Affiliations:** 1 Department of Physiology, University of California-San Francisco, San Francisco, 94158 California; 2 Howard Hughes Medical Institute, Chevy Chase, Maryland

**Keywords:** genome assembly, systems neuroscience, molecular neuroscience, neural plasticity, birdsong, Bengalese finch

## Abstract

**Background:**

Vocal learning in songbirds has emerged as a powerful model for sensorimotor learning. Neurobehavioral studies of Bengalese finch (*Lonchura striata domestica*) song, naturally more variable and plastic than songs of other finch species, have demonstrated the importance of behavioral variability for initial learning, maintenance, and plasticity of vocalizations. However, the molecular and genetic underpinnings of this variability and the learning it supports are poorly understood.

**Findings:**

To establish a platform for the molecular analysis of behavioral variability and plasticity, we generated an initial draft assembly of the Bengalese finch genome from a single male animal to 151× coverage and an N50 of 3.0 MB. Furthermore, we developed an initial set of gene models using RNA-seq data from 8 samples that comprise liver, muscle, cerebellum, brainstem/midbrain, and forebrain tissue from juvenile and adult Bengalese finches of both sexes.

**Conclusions:**

We provide a draft Bengalese finch genome and gene annotation to facilitate the study of the molecular-genetic influences on behavioral variability and the process of vocal learning. These data will directly support many avenues for the identification of genes involved in learning, including differential expression analysis, comparative genomic analysis (through comparison to existing avian genome assemblies), and derivation of genetic maps for linkage analysis. Bengalese finch gene models and sequences will be essential for subsequent manipulation (molecular or genetic) of genes and gene products, enabling novel mechanistic investigations into the role of variability in learned behavior.

## Introduction

Many motor skills, from walking and talking to the swing of a baseball bat, have the capacity for high degrees of both stability and flexibility between renditions. This capacity allows organisms to both reliably perform well-learned behaviors and to adapt behaviors in settings that present new environmental information. Regulation of this balance is a fundamental aspect of neural function, and its disruption may underlie neurological diseases characterized by excessive motor rigidity or variability, such as Parkinson's and Huntington's diseases [1,2]. Hence, understanding the neural mechanisms that mediate maintenance and adaptive modification of motor skills is critical to understanding the basis of both normal and pathological behavior.

The songs of songbirds are complex vocal motor skills and provide a powerful framework through which to understand the neural mechanisms that regulate motor skill learning, maintenance, and plasticity [3–5]. As with motor skills in humans, birdsong is learned and must be practiced to maintain performance. In particular, birdsong learning follows a similar developmental trajectory to human speech learning: song is initially acquired during an early critical period followed by a period of practice and then relatively invariant song production throughout adulthood [6]. Adult song relies on auditory feedback both to maintain song at a stable set point and to support adaptive change in response to environmental perturbations. Importantly, song production and learning is subserved by an anatomically discrete and functionally dedicated set of brain nuclei, which allows targeted characterization of electrophysiological and molecular properties of those nuclei that can be related back to song production, learning, and plasticity.

**Figure 1:**
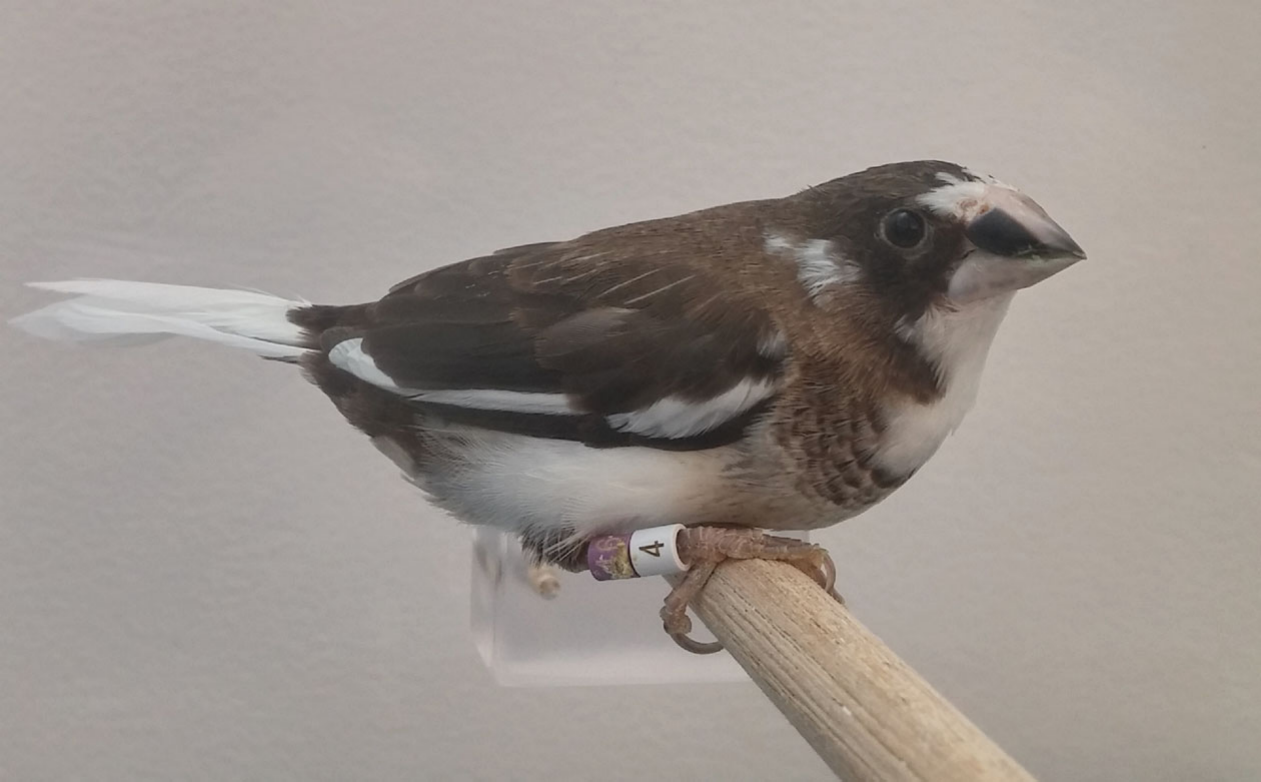
An adult male Bengalese finch (*Lonchura striata domestica*).

Relative to the songs of other commonly studied songbirds, the song of the Bengalese finch has several experimentally useful features that facilitate the study of behavioral variability in both learning and maintenance of complex behaviors. Bengalese finches (Fig. 1) exhibit substantial rendition-to-rendition variability in both the ordering and phonological attributes of their song elements [7]. This natural variation acts as a substrate for error-corrective and reinforcement learning [8–12] and has facilitated the analysis of how fluctuations in central nervous system activity lead to behavioral variation [13–15]. Furthermore, Bengalese finch song is more sensitive to auditory feedback and operant training paradigms than the songs of other songbird species. Complete loss of auditory feedback results in an increase in song sequence variability and rapid degradation of its spectral content [16,17]. Experiments using subtler distortions of auditory feedback indicate that Bengalese finches make corrections to adaptively adjust their song to minimize errors [9,18]. These studies, facilitated by behavior specific to the Bengalese finch, have provided insight into the neural mechanisms that drive variability and how that variability facilitates learning. However, studies of the molecular mechanisms that support this variability have been precluded by the absence of a genome assembly.

Beyond facilitating molecular studies of learning, this genome assembly is the first of a species in the genus *Lonchura*, which comprises approximately 37 species variously called munias or mannikins. Recent constructions of the Estrildid clade indicate that the *Lonchura* genus is monophyletic (with the exceptions of the African [*L. cantans*] and Indian [*L. malabarica*] silverbills) and radiated approximately 6 million years ago (MYA) [19–21]. The zebra finch (*Taenopygia guttata*), another commonly used model for vocal learning, shared a most recent common ancestor with the white-rumped munia approximately 9 MYA. The assembly provided here presents an opportunity for further comparative genomic work as well as molecular genetic analysis in a previously poorly studied genus.

The Bengalese finch is a domesticated variant of the white-rumped munia (*Lonchura striata*), an Estrildid finch that is indigenous to Southeast Asia including India, Myanmar, Thailand, Malaysia, and South China [22]. The birds are socially gregarious and live in large colonies that forage through open grasslands and urban backyards. The first well-documented case of domestication of the white-rumped munia is thought to have occurred approximately 250 years ago at the request of a Japanese feudal lord. Since then, the species has been selectively bred for tameness and reproductive efficiency [23]. Today, Bengalese finches (also known as Society finches) are widely kept as household pets. Interestingly, although there is no clear evidence that the Bengalese finch was bred for certain song characteristics, comparisons of the songs of the ancestral white-rumped munia and the Bengalese finch indicate that domestication has resulted in increased song complexity and a broader capacity to learn the songs of both the wild and domesticated variants [24,25]. Domestication has also led to laboratory populations that exhibit substantial interindividual variation in both plumage and song characteristics. The addition of a genome sequence for a domesticated species opens opportunities for comparative analysis into the impact of domestication on the genome.

Several songbird genome assemblies have been generated in recent years, including genomes for the zebra finch [26], canary [27], and American crow [28], opening up songbirds to genome-wide molecular analysis. However, the unique song features of Bengalese finches provide a system ideally suited to address specific questions regarding the molecular properties of the song system that facilitate or constrain song variability and the ability to respond to altered environmental conditions.

**Figure 2:**
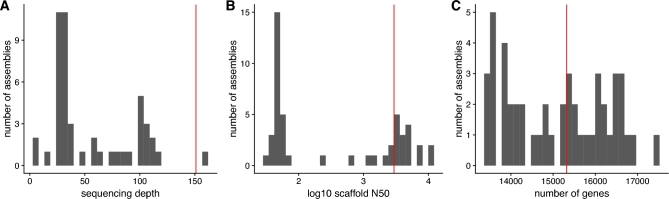
Comparison of Bengalese finch and Avian Phylogenomics Project assemblies. The distributions of sequencing depths (A), scaffold N50 (B), and number of annotated genes (C) are shown for the assemblies in the Avian Phylogenomics Project as of 14 September 2017. Vertical red line indicates the corresponding statistics for the Bengalese finch assembly and annotation described here.

To lay the groundwork for molecular studies in the Bengalese finch, we generated a high-coverage draft genome assembly and constructed an initial set of gene annotations. This assembly has coverage and scaffolding length that are on the upper ends of the distribution of assemblies in the Avian Phylogenomics Project [28] and has a comparable number of gene models (Fig. 2).

## Reuse potential

We expect that this resource will be used by other researchers for differential expression analysis, functional genomics, and comparative genomic analysis (through comparison to existing avian genomes), with a specific application to characterizing the differences between the genomes of the Bengalese finch and its ancestral species that contribute to differences in their songs [23]. The assembly can also be used as a reference for low-coverage sequencing and marker typing experiments that examine how genetic variation within a laboratory population contributes to heritable variation in song. Additionally, these gene models and sequences will be essential for manipulation (molecular or genetic) of genes and gene products, a prerequisite for developing models for molecular mechanisms. Moreover, this is the first large-scale genome assembly of a member of the *Lonchura* genus and will aid in further reconstructions of Estrildid phylogeny and in songbird evolution generally.

## Materials and Methods

### Animals

All birds were raised in our breeding colony at the University of California–San Francisco (UCSF). Experiments were conducted in accordance with National Institutes of Health and UCSF policies governing animal use and welfare (protocol number AN170723-01A).

### Genomic DNA library construction

Blood was collected from a single Bengalese finch adult male and purified using the DNeasy Blood & Tissue Kit (Qiagen).

We prepared 2 sets of libraries for genome assembly: one set with small insert size libraries and a second with larger insert size mate-pair libraries. First, small insert size libraries with 2 sizes were constructed. Two samples of 2.2 μg of genomic DNA were sonicated using a Covaris M220, 130 μL microTUBE, and presets for a target size of 200 bp (peak incident power 50 W, duty factor 20%, cycles per burst 200, treatment time 160 s). Samples were then purified using Sample Purification Beads (Illumina). Libraries were prepared from this sonicated gDNA using the TruSeq DNA PCR-Free LT Library Preparation Kit (Illumina). Briefly, samples were end repaired using End Repair Mix 2, then bead purified. Samples were then size selected using a BluePippin 2% agarose, dye-free, external marker gel (Sage Biosciences) set for 200 and 220 bp tight selection. Samples were then a-tailed, adapter ligated, and purified as indicated in the manufacturer's protocol.

Next, mate-pair libraries were constructed using the Nextera Mate-Pair Library Preparation Kit (Illumina) with 3, 5, and 9 kb insert sizes. Next, 4 μg purified genomic DNA was tagmented as recommended in the manufacturer's protocol, then purified using the Genomic DNA Clean and Concentrator Kit (Zymo). The protocol was continued through strand displacement and size selected using BluePippin 0.75% agarose, dye-free gels (broad selection at 2000–4000 bp, 4000–6000 bp, and 8000–10,000 bp, respectively). After selection, the protocol was continued through final polymerase chain reaction amplification.

### RNA collection and library construction

All tissues were dissected out, then minced and homogenized on ice. RNA was extracted using standard TRIzol extraction; 2 μg total RNA was DNase-treated using 2U rDNase I (Ambion) at 37°C for 25 minutes. DNase-treated total RNA was purified using RNA Clean and Concentrator 25 (Zymo), then 120 ng of this sample was prepared for sequencing using the Encore Complete DR RNA-seq Library System (NuGEN) according to the manufacturer's protocol. Table 1 provides tissue information including sex and ages of the animals.

**Table 1:** Descriptions of libraries used for genome assembly and gene annotation.

*Genomic libraries*					
Library	Insert size (expected)	Insert size (measured)	Reads (M)	Sequence (Gbases)	Coverage (x)
Fragment 1	200	202	403	50	42
Fragment 2	220	226	412	51	43
Jumping 1	3000	3300	753	60	50
Jumping 2	5000	5300	149	12	10
Jumping 3	9000	9000	100	7	6
Totals			1817	180	151

*RNA libraries*				
Tissue	Sex	Age (days post hatch)	Reads (M)	Sequence (Gbases)
Cerebellum	male	360	153	19
Forebrain	female	194	179	22
Forebrain	male	147	159	20
Forebrain	female	55	266	33
Forebrain	male	55	160	20
Liver	female	217	148	18
Midbrain/brainstem	male	360	182	23
Breast muscle	female	217	193	24
Totals			1439	180

### Sequencing

Small insert, mate-pair, and total RNA libraries were sequenced on 8 lanes of an Illumina HiSeq 2500 using V4 chemistry at Elim Biopharm (Hayward, California). Libraries were sequenced paired end to 125 cycles. Sequencing statistics are found in Table 1.

### Genome assembly

Sequencing data was assembled at the University of California–Davis Genome Center using ALLPATHS-LG (ALLPATHS-LG, RRID:SCR_010742) [29]. Prior to assembly, reads were trimmed for TruSeq (fragment libraries) or TruSeq and Nextera (jumping libraries) adapters using Trim Galore! [30], a wrapper for CutAdapt [31] and FastQC (FastQC, RRID:SCR_014583) [32]. TruSeq adaptor trimming was performed using: trim_galore –quality 20 -a AGATCGGAAGAG -a2 AGATCGGAAGAG –stringency 1. Nextera adaptor trimming was performed using: trim_galore –quality 20 -a CTGTCTCTTATA -a2 CTGTCTCTTATA –stringency 1. ALLPATHS-LG was then run using standard parameters. Statistics for the resulting assembly are provided in Table 2.

**Table 2:** Statistics of draft genome assembly

ALLPATHS-LG output	
Number of contigs	37 187
Number of contigs per Mb	35.1
Number of scaffolds	3016
Total contig length	1 027 319 005
Total scaffold length, with gap	1 058 688 097
N50 scaffold size in kb, with gaps	2953
Number of scaffolds per Mb	2.85
Median size of gaps in scaffolds	270
% of bases in captured gaps	2.94
**Assemblathon statistics**	
Total scaffold length as percentage of assumed genome size	88.30%
% of estimated genome that is useful (>= 25 kb)	87.60%
Longest scaffold	15 662 897
Shortest scaffold	887
Number of scaffolds > 1K nt	2987 (99.0%)
Number of scaffolds > 10K nt	1254 (41.6%)
Number of scaffolds > 100K nt	719 (23.8%)
Number of scaffolds > 1M nt	297 (9.8%)
Number of scaffolds > 10M nt	3 (0.1%)
Mean scaffold size	351 516
Median scaffold size	5349
N50 scaffold length	2 953 339
L50 scaffold count	103
NG50 scaffold length	2 494 006
LG50 scaffold count	129
N50 scaffold—NG50 scaffold length difference	459 333
Scaffold %A	28.31
Scaffold %C	20.13
Scaffold %G	20.09
Scaffold %T	28.24
Scaffold %N	2.94
Percentage of assembly in scaffolded contigs	99.60%
Percentage of assembly in unscaffolded contigs	0.40%
Average number of contigs per scaffold	10.5
Average length of break (>25 Ns) between contigs in scaffold	1082

### Repeat masking

The genome assembly was first masked for simple repeats and, using specific repeat models, generated using RepeatMasker open-4.0.5 [33] with -lib flag set using custom families generated using RepeatModeler open-1.0.8 [34]. Approximately 7.5% of the genome was classified as repetitive, comprising 80 Mbase of DNA. More detailed repeat element statistics can be found in Table 3.

**Table 3:** Repeat elements in the genome assembly identified by RepeatMasker

Class	N	Total length (Mbases)	Percent of genome
DNA	3460	0.31	0.03
LINE	118 051	32.03	3.03
Low_complexity	46 755	2.66	0.25
LTR	66 142	25.51	2.41
Satellite	3822	2.01	0.19
Simple_repeat	242 428	11.94	1.13
SINE	2163	0.15	0.01
Unknown	14 079	4.91	0.46
Total	496 900	79.52	7.52

### Transcript assembly and gene annotation

RNA library sequencing reads were first trimmed for TruSeq adapters using Trim Galore! (as above). Reads were aligned to the genome assembly using STAR v2.4.0h [35] set to remove noncanonical intron motifs (–outSAMstrandField intronMotif –outSAMattributes NH HI AS nM XS –outFilterIntronMotifs RemoveNoncanonical, otherwise default parameters), then assembled into transcripts using Cufflinks v2.2.1 (Cufflinks, RRID:SCR_014597) [36] (-j .5 –min-frags-per-transfrag 50 –max-intron-length 1 000 000, otherwise default parameters).

**Figure 3:**
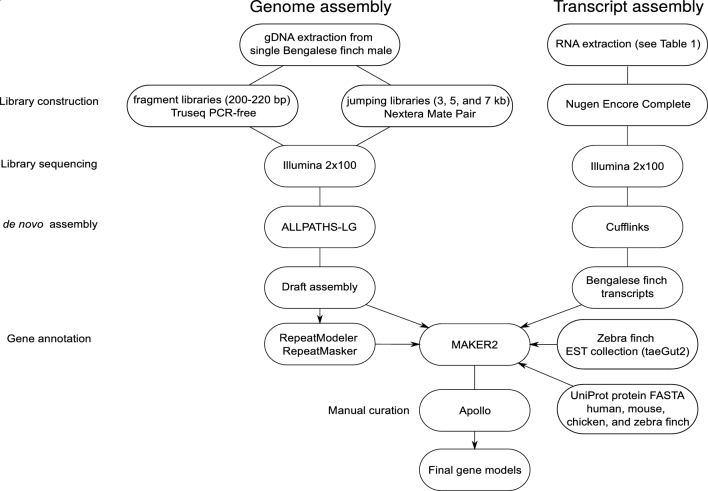
Flowchart of genome assembly and annotation. Experimental and computational approach used for genome assembly and gene annotation.

Gene annotation was performed using the MAKER2 pipeline [37] (Fig. 3). The following sources of evidence were used: Cufflinks transcript assembly described above; a collection of UniProt protein sequences from human, mouse, chicken, and zebra finch (each downloaded March 2, 2017); and Zebra finch EST collection (taeGut2) downloaded from UCSC the University of California, Santa Cruz Genome Browser (on 11 January 2015).

A random subset of gene models from the first MAKER2 run (n = 3859) was used to train Augustus v2.5.5 (Augustus: Gene Prediction, RRID:SCR_008417) [38], and the MAKER2 pipeline was rerun using these models to improve annotation. Next, 3^΄^ untranslated regions (UTRs) were added by intersecting these gene models with Cufflinks generated transcripts. MAKER2 generated 17 268 gene models that were filtered by AED scores below 0.5 (a measure of model support) to yield 15 313 models. All models were then manually curated as follows using Apollo v2.0.4 (Apollo, RRID:SCR_001936) [39]. Where possible, we corrected MAKER models that merged 2 genes, incorrectly split genes, or contained noncanonical splice junctions to eliminate frame shifts or truncated open reading frames and to best match aligned protein sequences. The 3^΄^ UTR positions were manually refined by selecting from the longest 3^΄^ UTR in the Cufflinks assembled transcripts without allowing overlaps between UTRs and adjacent genes on the same strand. These criteria were used to better facilitate read-gene assignment in 3^΄^ RNA-sequencing experiments. The most well-represented 5^΄^ UTRs were selected from the Cufflinks assembled transcripts. This curation yielded a set of 15 322 genes (the increase in gene number occurred due to splitting of some incorrectly merged genes and inclusion of well-supported genes from the Cufflinks transcript models that had been excluded by MAKER). Open reading frame sequences were aligned to the Uniprot-SwissProt protein database (downloaded 20 March 2015) using BLASTP [40] (default parameters except -max_target_seqs 1), which yielded 14 449 genes with a protein assignment with an e-value less than 10^−10^.

BUSCO (BUSCO, RRID:SCR_015008) [41], which detects near-universal single-copy orthologs to assay genome completeness, yielded 86% complete (n = 2621), 4% fragmented (n = 122), and 9% missing (n = 280) vertebrate genes (total n = 3023).

A comparison of this assembly and annotation with the assemblies in the Avian Phylogenomics Project can be found in Fig. 2. The full assembly and annotation were submitted to National Center for Biotechnology Information (NCBI) using custom scripts, GAG [42], Annie [43], and NCBI tbl2asn.

## Availability of supporting data

This Whole Genome Shotgun project has been deposited at DDBJ/ENA/GenBank under the accession MUZQ00000000. The version described in this paper is version MUZQ01000000. Supporting data, including transcriptome data, annotations, BUSCO results, and scripts are available via the *GigaScience* repository GigaDB [44].

## Abbreviations

MYA: million years ago; NCBI: National Center for Biotechnology Information; UCSF: University of California–San Francisco; UTR: untranslated region.

## Competing interests

All authors report no competing interests.

## Funding

This work was supported by the National Institute of Neurological Disorders and Stroke (F32NS098809) and the Howard Hughes Medical Institute.

## Author contributions

Bradley M. Colquitt designed the project, performed all experiments and analysis, and wrote the manuscript. David G. Mets and Michael S. Brainard conceived and designed the project.

## Supplementary Material

GIGA-D-17-00224_Original_Submission.pdf

GIGA-D-17-00224_Revision_1.pdf

Response_to_Reviewer_Comments_Original_Submission.pdf

Reviewer_1_Report_(Original_Submission) -- David Clayton 08 Nov 2017 Reviewed

Reviewer_2_Report_(Original_Submission) -- Morgan Wirthlin 23 Nov 2017 Reviewed

Reviewer_3_Report_(Original_Submission) -- Niclas Backström 26 Nov 2017 Reviewed
