# Supplementary material for: Draft genome assembly of the Bengalese finch, Lonchura striata domestica, a model for motor skill variability and learning
Source: Gigascience. 2018 Feb 15;7(3):giy008. doi: 10.1093/gigascience/giy008 (PMC5861438; doi:10.1093/gigascience/giy008)

GigaScience

Draft genome assembly of the Bengalese finch, Lonchura striata domestica, a model for motor skill variability and learning

--Manuscript Draft--

Manuscript Number:	GIGA-D-17-00224
Full Title:	Draft genome assembly of the Bengalese finch, Lonchura striata domestica, a model for motor skill variability and learning
Article Type:	Data Note
Funding Information:	Howard Hughes Medical Institute (US) (Investigator Award) — Dr Michael Scott Brainard<br>National Institute of Neurological Disorders and Stroke (F32NS098809) — Dr Bradley Mark Colquitt
Abstract:	Background:<br>The study of song learning in songbirds has emerged as a powerful model for sensorimotor learning. Neuro-behavioral studies of Bengalese finch (Lonchura striata domestica) song, naturally more variable and plastic than songs of other finch species, have demonstrated the importance of behavioral variability for initial learning, maintenance, and plasticity of vocalizations. However, the molecular and genetic underpinnings of this variability, and the learning it supports, are poorly understood.<br><br>Findings:<br>To establish a platform for the molecular analysis of song variability and plasticity, we have generated an initial draft assembly of the Bengalese finch genome from a single male animal to 151x coverage and an N50 of 3.0 MB. Furthermore, we have developed an initial set of gene models using RNA-seq data from eight samples that comprise liver, muscle, cerebellum, brainstem/midbrain and forebrain tissue from juvenile and adult Bengalese finches of both sexes.<br><br>Conclusions:<br>We provide a draft Bengalese finch genome and gene annotation to facilitate the study of the molecular-genetic influences on behavioral variability and the process of vocal learning. These data will directly support many avenues for the identification of genes involved in learning, including differential expression analysis, comparative genomic analysis (through comparison to extant avian genomes), and derivation of genetic maps for linkage analysis. Bengalese finch gene models and sequences will be essential for subsequent manipulation (molecular or genetic) of genes and gene products, enabling novel mechanistic investigations into the role of variability in learned behavior.
Corresponding Author:	Bradley Mark Colquitt, Ph.D.<br>University of California San Francisco<br>San Francisco, CA UNITED STATES
Corresponding Author Secondary Information:	
Corresponding Author's Institution:	University of California San Francisco
Corresponding Author's Secondary Institution:	
First Author:	Bradley Mark Colquitt, Ph.D.
First Author Secondary Information:	
Order of Authors:	Bradley Mark Colquitt, Ph.D.
	David G Mets, Ph.D.
	Michael Scott Brainard, Ph.D.
Order of Authors Secondary Information:	

Powered by Editorial Manager® and ProduXion Manager® from Aries Systems Corporation

**Opposed Reviewers:**	
**Additional Information:**	
**Question**	**Response**
Are you submitting this manuscript to a special series or article collection?	No
**Experimental design and statistics**<br><br>Full details of the experimental design and statistical methods used should be given in the Methods section, as detailed in our Minimum Standards Reporting Checklist. Information essential to interpreting the data presented should be made available in the figure legends.<br><br>Have you included all the information requested in your manuscript?	Yes
**Resources**<br><br>A description of all resources used, including antibodies, cell lines, animals and software tools, with enough information to allow them to be uniquely identified, should be included in the Methods section. Authors are strongly encouraged to cite Research Resource Identifiers (RRIDs) for antibodies, model organisms and tools, where possible.<br><br>Have you included the information requested as detailed in our Minimum Standards Reporting Checklist?	Yes
**Availability of data and materials**<br><br>All datasets and code on which the conclusions of the paper rely must be either included in your submission or deposited in publicly available repositories (where available and ethically appropriate), referencing such data using a unique identifier in the references and in the “Availability of Data and Materials” section of your manuscript.<br><br>Have you have met the above requirement as detailed in our Minimum Standards Reporting Checklist?	Yes

Powered by Editorial Manager® and ProduXion Manager® from Aries Systems Corporation

Manuscript Click here to download Manuscript data_description_16.pdf

Draft genome assembly of the Bengalese finch, *Lonchura striata domestica,* a model for motor skill variability and learning

Bradley M. Colquitt[1,*] bradley.colquitt@ucsf.edu

David G. Mets[1] dmets@phy.ucsf.edu

Michael S. Brainard[1,2] msb@phy.ucsf.edu

1. Department of Physiology, University of California-San Francisco, San Francisco, California, USA

2. Howard Hughes Medical Institute, Chevy Chase, Maryland, USA

* Corresponding author

Abstract

Background:

Vocal learning in songbirds has emerged as a powerful model for sensorimotor learning. Neuro-behavioral studies of Bengalese finch (*Lonchura striata domestica*) song, naturally more variable and plastic than songs of other finch species, have demonstrated the importance of behavioral variability for initial learning, maintenance, and plasticity of vocalizations. However, the molecular and genetic underpinnings of this variability, and the learning it supports, are poorly understood.

Findings:

To establish a platform for the molecular analysis of behavioral variability and plasticity, we have generated an initial draft assembly of the Bengalese finch genome from a single male animal to 151x coverage and an N50 of 3.0 MB. Furthermore, we have developed an initial set of gene models using RNA-seq data from eight samples that comprise liver, muscle, cerebellum, brainstem/midbrain and forebrain tissue from juvenile and adult Bengalese finches of both sexes.

Conclusions:

We provide a draft Bengalese finch genome and gene annotation to facilitate the study of the molecular-genetic influences on behavioral variability and the process of vocal learning. These data will directly support many avenues for the identification of genes involved in learning, including differential expression analysis, comparative genomic analysis (through comparison to existing avian genome assemblies), and derivation of genetic maps for linkage analysis. Bengalese finch gene models and sequences will be essential for subsequent manipulation (molecular or genetic) of genes and gene products, enabling novel mechanistic investigations into the role of variability in learned behavior.

Keywords

Genome assembly, systems neuroscience, molecular neuroscience, neural plasticity, birdsong, Bengalese finch

Data Description

Many motor skills, from walking and talking to the swing of a baseball bat, have the capacity for high degrees of both stability and flexibility between renditions. This capacity allows organisms to both reliably perform well-learned behaviors and to adapt behaviors in settings that present new environmental information. Regulation of this balance is a fundamental aspect of neural function, and its disruption may underlie neurological diseases characterized by excessive motor rigidity or variability, such as Parkinson's and Huntington's diseases [1,2]. Hence, understanding the neural mechanisms that mediate maintenance and adaptive modification of motor skills is critical to understanding the basis of both normal and pathological behavior.

The songs of songbirds are complex vocal motor skills and provide a powerful framework through which to understand the neural mechanisms that regulate motor skill learning, maintenance, and

plasticity [3–5]. As with motor skills in humans, birdsong is learned and must be practiced to maintain performance. In particular, birdsong learning follows a similar developmental trajectory to human speech learning: song is initially acquired during an early critical period followed by a period of practice and then relatively invariant song production throughout adulthood [6]. Adult song relies on auditory feedback both to maintain song at a stable setpoint and to support adaptive change in response to environmental perturbations. Importantly, song production and learning is subserved by an anatomically discrete and functionally dedicated set of brain nuclei, which allows targeted characterization of electrophysiological and molecular properties of those nuclei that can be related back to song production, learning, and plasticity.

Relative to the songs of other commonly studied songbirds, the song of the Bengalese finch has several experimentally useful features that facilitate the study of behavioral variability in both learning and maintenance of complex behaviors. Bengalese finches (Fig. 1) exhibit substantial rendition-to-rendition variability in both the ordering and phonological attributes of their song elements [7]. This natural variation acts as a substrate for error-corrective learning [8–11] and has facilitated the analysis of how fluctuations in central nervous system activity lead to behavioral variation [12,13]. Furthermore, Bengalese finch song is more sensitive to auditory feedback and operant training paradigms than the songs of other songbird species. Complete loss of auditory feedback results in an increase in song sequence variability and the rapid degradation of its spectral content [14,15]. Experiments using subtler distortions of auditory feedback indicate that Bengalese finches make corrections to adaptively adjust their song to minimize errors [9,16]. These studies, facilitated by behavior specific to the Bengalese finch, have provided insight into the neural mechanisms driving variability and how that variability facilitates learning. However, studies of the molecular mechanisms which support this variability have been precluded by the absence of a genome assembly.

Beyond facilitating molecular studies of learning, this genome assembly is the first of a species in the genus *Lonchura,* which comprises approximately 37 species variously called munias or mannikins. Recent constructions of the Estrildid clade indicate that the *Lonchura* genus is monophyletic (with the exceptions of the African (*L. cantans*) and Indian (*L. malabarica*) silverbills) and radiated approximately 6 million years ago (MYA) [17–19]. The zebra finch (*Taenopygia guttata*), which is also an Estrildid and has the mostly completely assembled songbird genome to date, shared a most recent common ancestor with the white-rumped munia ~9 MYA. The assembly provided here presents an opportunity for further comparative genomic work as well as molecular genetic analysis in a previously poorly studied genus.

Bengalese finches are a domesticated variant of the white-rumped munia (*Lonchura striata),* an Estrildid finch that is indigenous to Southeast Asia including India, Myanmar, Thailand, Malaysia, and South China [20]. The birds are socially gregarious and live in large colonies that forage through open grasslands and urban backyards. The first well-documented case of domestication of the white-rumped munia is thought to have occurred approximately 250 years ago at the request of a Japanese feudal lord, and the species has been selectively bred for tameness and reproductive efficiency [21]. Today, Bengalese finches (also known as Society finches) are widely kept as household pets. Interestingly, although there is no clear evidence that the Bengalese finch was bred for certain song characteristics, comparisons of the songs of the ancestral white-rumped munia and the Bengalese finch indicate that domestication has resulted in increased song complexity and a broader capacity to learn the songs of both the wild and domesticated variants [22,23]. Domestication has also led to laboratory populations that exhibit substantial interindividual variation in both plumage and song characteristics. This phenotypic variation is matched by high levels of genetic variation. Marker typing analysis of our outbred colony indicates polymorphism densities on the order of outbred human populations [D. Mets and M. Brainard, unpublished observation]. The addition of a genome sequence

for a domesticated species opens opportunities for comparative analysis into the impact of domestication on the genome.

Several songbird genome assemblies have been generated in recent years, including genomes for the zebra finch [24], canary [25], and American crow [26], opening up songbirds to genome-wide molecular analysis. However, the unique song features of Bengalese finches provide a system ideally suited to address specific questions regarding the molecular properties of the song system that facilitate or constrain song variability and the ability to respond to altered environmental conditions.

To lay the groundwork for molecular studies in the Bengalese finch, we generated a high coverage draft genome assembly and constructed an initial set of gene annotations.

Re-use potential

We expect that this resource will be used by other researchers for differential expression analysis, functional genomics, and comparative genomic analysis (through comparison to existing avian genomes), with a specific application to characterizing the differences between the genomes of the Bengalese finch and its ancestral species that contribute to differences in their songs [21]. The assembly can also be used as a reference for low coverage sequencing and marker typing experiments examining how genetic variation within a laboratory populations contributes to heritable variation in song. Additionally, these gene models and sequences will be essential for manipulation (molecular or genetic) of genes and gene products, a prerequisite for developing models for molecular mechanisms. Moreover, this is first large-scale genome assembly of a member of the *Lonchura* genus and will aid in further reconstructions of Estrildid phylogeny and in songbird evolution generally.

Methods

Animals

All birds were from our breeding colony at UCSF, and experiments were conducted in accordance with NIH and UCSF policies governing animal use and welfare.

Genomic DNA library construction

Blood was collected from a single Bengalese finch adult male and purified using DNeasy Blood & Tissue Kit (Qiagen).

We prepared two sets of libraries for genome assembly: one set with small insert size libraries and a second with larger insert size mate-pair libraries. First, small insert size libraries with two different sizes were constructed. Two samples of 2.2 ug of genomic DNA were sonicated using a Covaris M220, 130 µl microTUBE, and presets for a target size of 200 bp (peak incident power 50 W, duty factor 20%, cycles per burst 200, treatment time 160 s). Samples were then purified using Sample Purification Beads (Illumina). Libraries were prepared from this sonicated gDNA using the TruSeq DNA PCR-Free LT Library Preparation Kit (Illumina). Briefly, samples were end repaired using End Repair Mix 2 then bead purified. Samples were then size selected using a BluePippin 2% agarose, dye-free, external marker gel (Sage Biosciences) set for 200 and 220 bp tight selection. Samples were then a-tailed, adapter ligated, and purified as indicated in the manufacturer's protocol.

Next, mate-pair libraries were constructed using the Nextera Mate-Pair Library Preparation Kit (Illumina) with 3, 5, and 9 kb insert sizes. 4 µg purified genomic DNA was tagmented as recommended in the manufacturer's protocol then purified using the Genomic DNA Clean and Concentrator Kit (Zymo). The protocol was continued through strand displacement and size selected using BluePippin 0.75% agarose, dye-free gels (broad selection at 2000-4000 bp, 4000-6000 bp, and 8000-10,000 bp respectively). After selection, the protocol was continued through final PCR amplification.

RNA collection and library construction

All tissues were dissected out then minced and homogenized on ice. RNA was extracted using standard TRIzol extraction. 2 μg total RNA was DNase-treated using 2U rDNase I (Ambion) at 37°C for 25 minutes. DNase-treated total RNA was purified using RNA Clean and Concentrator 25 (Zymo) then 120 ng of this sample was prepared for sequencing using the Encore Complete DR RNA-seq Library System (NuGEN) according to the manufacturer's protocol. Table 1 provides tissue information including sex and ages of the animals.

Sequencing

Small insert, mate-pair, and total RNA libraries were sequenced on eight lanes of an Illumina HiSeq 2500 using V4 chemistry at Elim Biopharm (Hayward, CA). Libraries were sequenced paired end to 125 cycles. Sequencing statistics are found in Table 1.

Genome assembly

Sequencing data was assembled at the UC Davis Genome Center using ALLPATHS-LG [27]. Prior to assembly, reads were trimmed for TruSeq (fragment libraries) or Nextera (jumping libraries) adapters using Trim Galore!, a wrapper for CutAdapt [28] and FastQC [29]. ALLPATHS-LG was then run using standard parameters. Statistics for the resulting assembly are in Table 2.

Repeat masking

The genome assembly was first masked for simple repeats and using specific repeat models generated using RepeatMasker open-4.0.5 [30] with -lib flag set using custom families generated using RepeatModeler open-1.0.8 [31]. Approximately 7.5% of the genome was classified as repetitive, comprising 80 Mbase of DNA. More detailed repeat element statistics can be found in Table 3.

Transcript assembly and gene annotation

RNA library sequencing reads were first trimmed for TruSeq adapters and poly-adenosine tails using Trim Galore!. Reads were aligned to the genome assembly using STAR v2.4.0h [32] set to remove non-canonical intron motifs, then assembled into transcripts using Cufflinks v2.2.1 [33] (-j .5 –min-frags-per-transfrag 50 –max-intron-length 1000000, otherwise default parameters).

Gene annotation was performed using the MAKER2 pipeline [34] (Fig. 2). The following sources of evidence were used:

1) Cufflinks transcript assembly described above
2) A collection of UniProt protein sequences from human, mouse, chicken, and zebra finch.
3) Zebra finch EST collection (taeGut2) downloaded from UCSC.

An initial set of gene models was used to train Augustus v2.5.5 [35], and the MAKER2 pipeline was re-run using these models to improve annotation. 3' UTRs were added by intersecting these gene models with Cufflinks generated transcripts. MAKER2 generated 17,268 gene models that were filtered by AED scores below 0.5 (a measure of model support) to yield 15,313 models. These models were then manually curated using Apollo v2.0.4 [36] to ensure completeness and to refine UTR positions. Open reading frame sequences were aligned to the Uniprot-SwissProt protein database (downloaded 3/20/2015) using BLASTP [37], which yielded 14,449 genes with a protein assignment with e-value less than 10^{-10}.

CEGMA [38], which detects highly conserved genes (CEGs) to assay genome completeness, yielded 65% complete CEGs and 94% partial CEGs. A similar approach, BUSCO [39], which detects near-universal single-copy orthologs, yielded 86% complete (n=2621), 4% fragmented (n=122), and 9% missing (n=280) vertebrate genes (total n=3023).

A comparison of this assembly and annotation with the assemblies in the Avian Phylogenomics Project can be found in Figure 3. The full assembly and annotation were submitted to NCBI using custom scripts, GAG [40], Annie [41], and NCBI tbl2asn.

The authors declare that they have no competing interests.

Availability of data

This Whole Genome Shotgun project has been deposited at DDBJ/ENA/GenBank under the accession MUZQ00000000. The version described in this paper is version MUZQ01000000. Supporting data is also available in the *GigaScience* database [see FTP server during review process].

Funding

This work was supported by the National Institute of Neurological Disorders and Stroke (F32NS098809) and the Howard Hughes Medical Institute.

Authors' contributions

BC designed the project, performed all experiments and analysis, and wrote the manuscript. DM and MB conceived and designed the project.

Acknowledgements

We thank Dr. Joe Fass and Richard Feltstykeet from the UC Davis Genome Center Bioinformatics Core for their tremendous help and consultation which contributed to the success of this project. We also thank Foad Green for his help manually curating the gene annotation.

References

1. Wolpert DM, Diedrichsen J, Flanagan JR. Principles of sensorimotor learning. Nat. Rev. Neurosci. 2011;12:739–51.

2. Doyon J. Motor sequence learning and movement disorders. Curr. Opin. Neurol. 2008;21:478–83.

3. Brainard MS, Doupe AJ. What songbirds teach us about learning. Nature. 2002;417:351–8.

4. Brainard MS, Doupe AJ. Translating birdsong: songbirds as a model for basic and applied medical research. Annu. Rev. Neurosci. 2013;36:489–517.

5. Konishi M. Birdsong for neurobiologists. Neuron. 1989;3:541–9.

6. Doupe AJ, Kuhl PK. Birdsong and human speech: common themes and mechanisms. Annu. Rev. Neurosci. 1999;22:567–631.

7. Okanoya K. The Bengalese Finch: A Window on the Behavioral Neurobiology of Birdsong Syntax. Ann. N. Y. Acad. Sci. [Internet]. 2004 [cited 2017 Jul 27];1016:724–35. Available from: http://doi.wiley.com/10.1196/annals.1298.026

8. Tumer EC, Brainard MS. Performance variability enables adaptive plasticity of “crystallized” adult birdsong. Nature. 2007;450:1240–4.

9. Sober SJ, Brainard MS. Adult birdsong is actively maintained by error correction. Nat. Neurosci. [Internet]. 2009;12:927–31. Available from: http://dx.doi.org/10.1038/nn.2336

10. Warren TL, Tumer EC, Charlesworth JD, Brainard MS. Mechanisms and time course of vocal learning and consolidation in the adult songbird. J. Neurophysiol. 2011;106:1806–21.

11. Warren TL, Charlesworth JD, Tumer EC, Brainard MS. Variable sequencing is actively maintained in a well learned motor skill. J. Neurosci. 2012;32:15414–25.

12. Sober SJ, Wohlgemuth MJ, Brainard MS. Central contributions to acoustic variation in birdsong. J. Neurosci. 2008;28:10370–9.

13. Fujimoto H, Hasegawa T, Watanabe D. Neural Coding of Syntactic Structure in Learned Vocalizations in the Songbird. J. Neurosci. 2011;31.

14. Okanoya K, Yamaguchi A. Adult Bengalese finches (Lonchura striata var. domestica) require real-time auditory feedback to produce normal song syntax. J. Neurobiol. 1997;33:343–56.

15. Woolley SM, Rubel EW. Bengalese finches Lonchura Striata domestica depend upon auditory feedback for the maintenance of adult song. J. Neurosci. 1997;17:6380–90.

16. Sakata JT, Brainard MS. Real-time contributions of auditory feedback to avian vocal motor control. J. Neurosci. [Internet]. Society for Neuroscience; 2006;26:9619–28. Available from: http://www.jneurosci.org/content/26/38/9619.full

17. Hooper DM, Price TD. Rates of karyotypic evolution in Estrildid finches differ between island and continental clades. Evolution (N. Y). [Internet]. 2015 [cited 2017 Sep 8];69:890–903. Available from: http://doi.wiley.com/10.1111/evo.12633

18. Arnaiz-Villena A, Ruiz-Del-Valle V, Gomez-Prieto P, Reguera R, Parga-Lozano C, Serrano-Vela I. Estrildinae Finches (Aves, Passeriformes) from Africa, South Asia and Australia: a Molecular

Phylogeographic Study. Open Ornithol. J. [Internet]. 2009 [cited 2017 Sep 8];2:29–36. Available from: http://chopo.pntic.mec.es/biolmol/publicaciones/Estrildinae_finches_2009.pdf

19. Sorenson MD, Balakrishnan CN, Payne RB, Johnson K. Clade-Limited Colonization in Brood Parasitic Finches (Vidua spp.). Syst. Biol. [Internet]. Sinauer, Sunderland, Massachusetts; 2004 [cited 2017 Sep 8];53:140–53. Available from: http://academic.oup.com/sysbio/article/53/1/140/2842856/CladeLimited-Colonization-in-Brood-Parasitic

20. Restall R. Munias and Mannikins. East Sussex, UK: Pica Press; 1996.

21. Okanoya K. Evolution of song complexity in Bengalese finches could mirror the emergence of human language. J. Ornithol. [Internet]. 2015 [cited 2017 Jul 27];156:65–72. Available from: http://link.springer.com/10.1007/s10336-015-1283-5

22. Honda E, Okanoya K. Acoustical and Syntactical Comparisons between Songs of the White-backed Munia (Lonchura striata) and Its Domesticated Strain, the Bengalese Finch (Lonchura striata var. domestica). Zoolog. Sci. [Internet]. 1999 [cited 2014 Jul 14];16:319–26. Available from: http://dx.doi.org/10.2108/zsj.16.319

23. Takahasi M, Okanoya K. Song Learning in Wild and Domesticated Strains of White-Rumped Munia, Lonchura striata , Compared by Cross-Fostering Procedures: Domestication Increases Song Variability by Decreasing Strain-Specific Bias. Ethology [Internet]. 2010 [cited 2017 Jul 27];116:396–405. Available from: http://doi.wiley.com/10.1111/j.1439-0310.2010.01761.x

24. Warren WC, Clayton DF, Ellegren H, Arnold AP, Hillier LW, Künstner A, et al. The genome of a songbird. Nature. 2010;464:757–62.

25. Frankl-Vilches C, Kuhl H, Werber M, Klages S, Kerick M, Bakker A, et al. Using the canary genome to decipher the evolution of hormone-sensitive gene regulation in seasonal singing birds. Genome Biol. 2015;16:19.

26. Zhang G, Li B, Li C, Gilbert MTP, Jarvis ED, Wang J. Comparative genomic data of the Avian Phylogenomics Project. Gigascience. 2014;3:26.

27. Gnerre S, Maccallum I, Przybylski D, Ribeiro FJ, Burton JN, Walker BJ, et al. High-quality draft assemblies of mammalian genomes from massively parallel sequence data. Proc. Natl. Acad. Sci. U. S. A. 2011;108:1513–8.

28. Martin M. Cutadapt removes adapter sequences from high-throughput sequencing reads. EMBnet.journal. 2011;17:10.

29. Andrews S. FastQC [Internet]. 2015. Available from: http://www.bioinformatics.bbsrc.ac.uk/projects/fastqc

30. Smit A, Hubley R, Green P. RepeatMasker Open-4.0 [Internet]. 2013. Available from: http://www.repeatmasker.org

31. Smit AFA, Hubley R. RepeatModeler Open-1.0 [Internet]. 2010. Available from: http://www.repeatmasker.org

32. Dobin A, Davis CA, Schlesinger F, Drenkow J, Zaleski C, Jha S, et al. STAR: ultrafast universal RNA-seq aligner. Bioinformatics. 2013;29:15–21.

33. Roberts A, Pimentel H, Trapnell C, Pachter L. Identification of novel transcripts in annotated genomes using RNA-Seq. Bioinformatics. 2011;27:2325–9.

34. Holt C, Yandell M. MAKER2: an annotation pipeline and genome- database management tool for second- generation genome projects. BMC Bioinformatics. 2011;12.

35. Stanke M, Keller O, Gunduz I, Hayes A, Waack S, Morgenstern B. AUGUSTUS: ab initio prediction of alternative transcripts. Nucleic Acids Res. [Internet]. 2006 [cited 2017 Jan 30];34:W435-9. Available from: http://www.ncbi.nlm.nih.gov/pubmed/16845043

36. Lee E, Helt GA, Reese JT, Munoz-Torres MC, Childers CP, Buels RM, et al. Web Apollo: a web-based genomic annotation editing platform. Genome Biol. 2013;14.

37. Camacho C, Coulouris G, Avagyan V, Ma N, Papadopoulos J, Bealer K, et al. BLAST+: architecture and applications. BMC Bioinformatics. 2009;10:421.

38. Parra G, Bradnam K, Korf I. CEGMA: a pipeline to accurately annotate core genes in eukaryotic genomes. Bioinformatics. 2007;23:1061–7.

39. Simão FA, Waterhouse RM, Ioannidis P, Kriventseva E V., Zdobnov EM, S.C. C, et al. BUSCO: assessing genome assembly and annotation completeness with single-copy orthologs. Bioinformatics. 2015;31:3210–2.

40. Hall B, DeRego T, Geib S. GAG: the Genome Annotation Generator (Version 1.0) [Internet]. 2014. Available from: http://genomeannotation.github.io/GAG

41. Tate R, Hall B, DeRego T, Geib S. Annie: the ANNotation Information Extractor (Version 1.0) [Internet]. 2014. Available from: http://genomeannotation.github.io/annie

Figure legends

Figure 1. Bengalese finch (*Lonchura striata domestica*). An adult male Bengalese finch.

Figure 2. Flowchart of genome assembly and annotation. Experimental and computational approach used for genome assembly and gene annotation.

Figure 3. Comparison of Bengalese finch and Avian Phylogenomics Project assemblies. The distributions o sequencing depths (A), scaffold N50 (B), and number of annotated genes (C) are show for the assemblies in the Avian Phylogenomics Project as of September 14, 2017. Vertical red line indicates the corresponding statistics for the Bengalese finch assembly and annotation described here.

Table legends

Table 1. Descriptions of libraries used for genome assembly and gene annotation.

Table 2. Statistics of draft genome assembly.

Table 3. Repeat elements in the genome assembly identified by RepeatMasker.

Tables

Table 1 Click here to download Table table1.pdf

Tables

Table 1. Sequencing libraries

Genomic libraries

Library	Insert size expected	Insert size measured	Reads (M)	Sequence (Gbases)	Coverage (x)
Fragment 1	200	202	403	50	42
Fragment 2	220	226	412	51	43
Jumping 1	3000	3300	753	60	50
Jumping 2	5000	5300	149	12	10
Jumping 3	9000	9000	100	7	6
Totals			1817	180	151

RNA libraries

Tissue	Sex	Age	Reads (M)	Sequence (Gbases)
Forebrain	female	194	179	22
Forebrain	male	147	159	20
Muscle	female	217	193	24
Liver	female	217	148	18
Cerebellum	male	360	153	19
Midbrain/brain	male	360	182	23
Forebrain	female	55	266	33
Forebrain	male	55	160	20
Totals			1439	180

Table 2 Click here to download Table table2.pdf

Table 2. Assembly statistics

ALLPATHS-LG output	
number of contigs	37187
number of contigs per Mb	35.1
number of scaffolds	3016
total contig length	1027319005
total scaffold length, with gap	1058688097
N50 scaffold size in kb, with gaps	2953
number of scaffolds per Mb	2.85
median size of gaps in scaffolds	270
% of bases in captured gaps	2.94

Assemblathon statistics	
Total scaffold length as percentage of assumed genome size	88.30%
% of estimated genome that is useful (>= 25 kb)	87.60%
Longest scaffold	15662897
Shortest scaffold	887
Number of scaffolds > 1K nt	2987 (99.0%)
Number of scaffolds > 10K nt	1254 (41.6%)
Number of scaffolds > 100K nt	719 (23.8%)
Number of scaffolds > 1M nt	297 (9.8%)
Number of scaffolds > 10M nt	3 (0.1%)
Mean scaffold size	351516
Median scaffold size	5349
N50 scaffold length	2953339
L50 scaffold count	103
NG50 scaffold length	2494006
LG50 scaffold count	129
N50 scaffold - NG50 scaffold length difference	459333
scaffold %A	28.31
scaffold %C	20.13
scaffold %G	20.09
scaffold %T	28.24
scaffold %N	2.94
Percentage of assembly in scaffolded contigs	99.60%
Percentage of assembly in unscaffolded contigs	0.40%
Average number of contigs per scaffold	10.5
Average length of break (>25 Ns) between contigs in scaffold	1082

338

Table 3. Repeat element statistics

Class	N	Total length (Mbases)	Percent of genome
DNA	3460	0.31	0.03
LINE	118051	32.03	3.03
Low_complexity	46755	2.66	0.25
LTR	66142	25.51	2.41
Satellite	3822	2.01	0.19
Simple_repeat	242428	11.94	1.13
SINE	2163	0.15	0.01
Unknown	14079	4.91	0.46
Total	496900	79.52	7.52

Figure 1. Bengalese finch (*Lonchura striata domestica)*

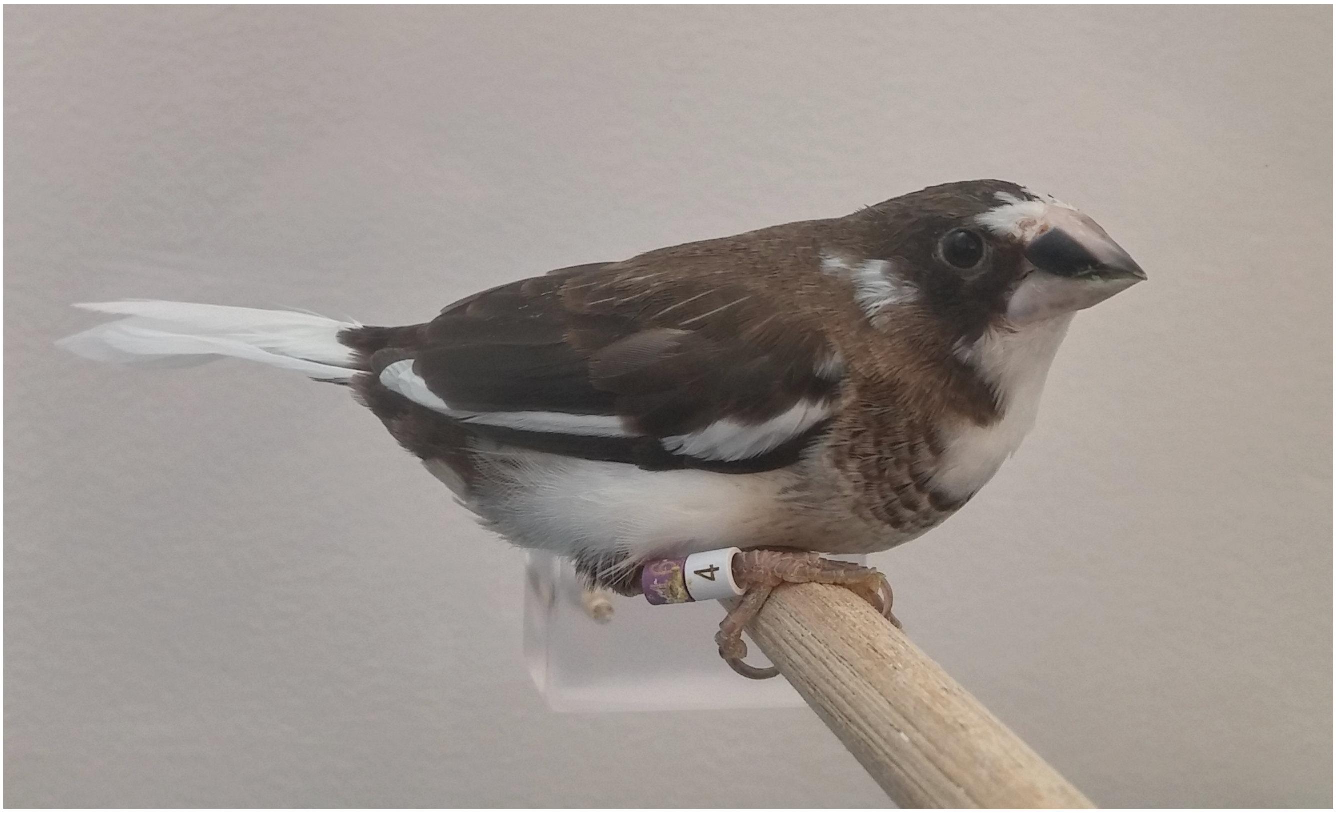

Figure 2. Flowchart of genome assembly and annotation.

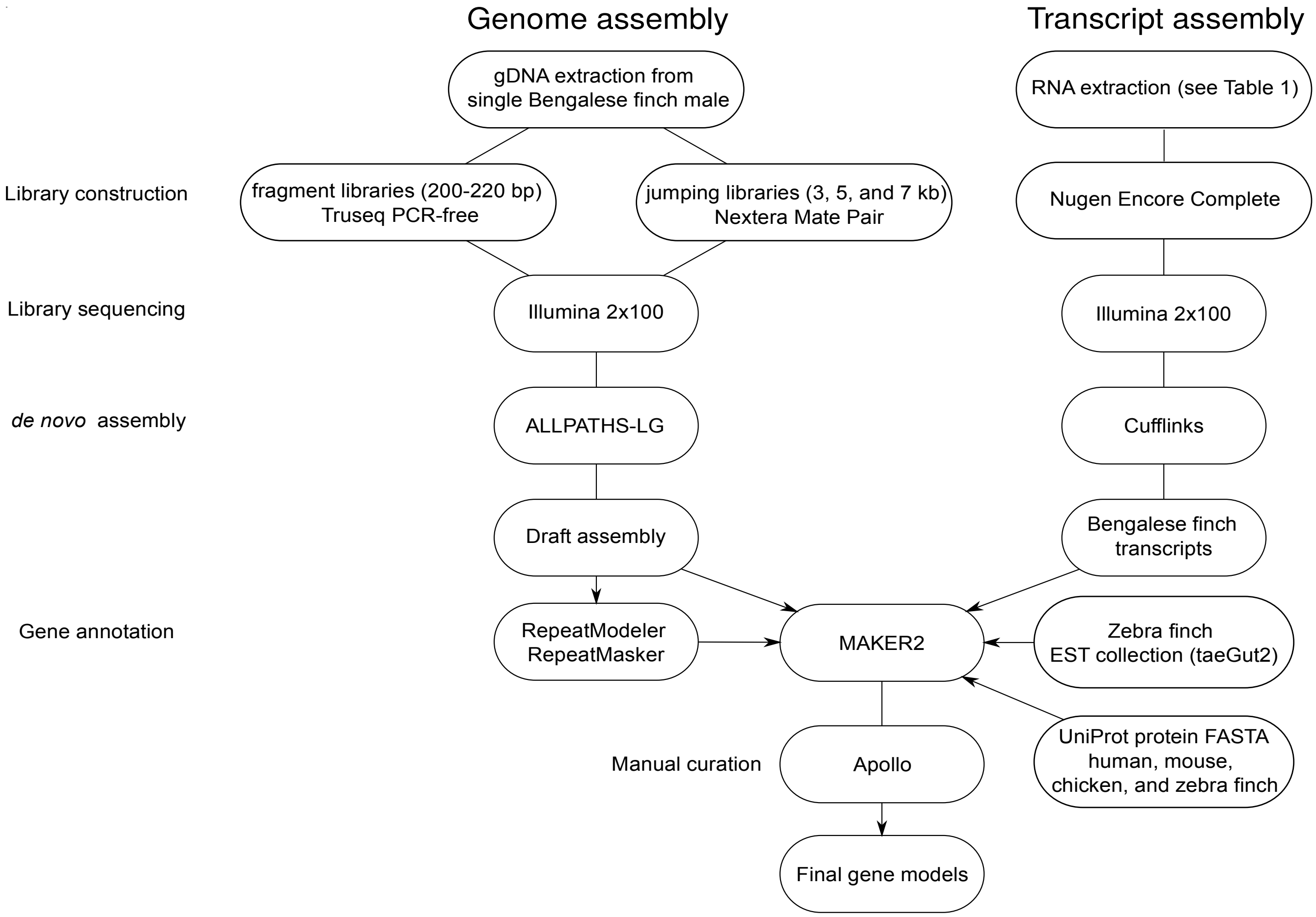

Figure3

Click here to download Figure figure3.pdf

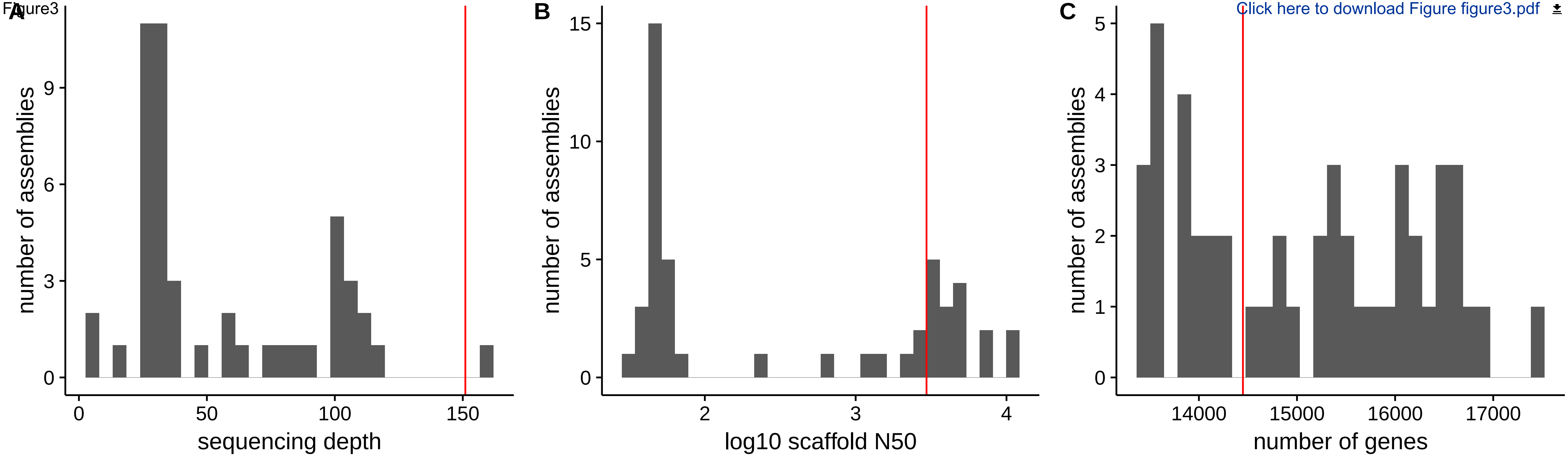

Supplement: GIGA-D-17-00224_Original_Submission.pdf [file giy008_giga-d-17-00224_original_submission.pdf]
