# Supplementary material for: Draft genome assembly of the Bengalese finch, Lonchura striata domestica, a model for motor skill variability and learning
Source: Gigascience. 2018 Feb 15;7(3):giy008. doi: 10.1093/gigascience/giy008 (PMC5861438; doi:10.1093/gigascience/giy008)

GigaScience

Draft genome assembly of the Bengalese finch, Lonchura striata domestica, a model for motor skill variability and learning

--Manuscript Draft--

**Manuscript Number:**	GIGA-D-17-00224R1
**Full Title:**	Draft genome assembly of the Bengalese finch, Lonchura striata domestica, a model for motor skill variability and learning
**Article Type:**	Data Note
**Funding Information:**	Howard Hughes Medical Institute (US) (Investigator Award) — Dr Michael Scott Brainard<br>National Institute of Neurological Disorders and Stroke (F32NS098809) — Dr Bradley Mark Colquitt
**Abstract:**	Background:<br>The study of song learning in songbirds has emerged as a powerful model for sensorimotor learning. Neuro-behavioral studies of Bengalese finch (Lonchura striata domestica) song, naturally more variable and plastic than songs of other finch species, have demonstrated the importance of behavioral variability for initial learning, maintenance, and plasticity of vocalizations. However, the molecular and genetic underpinnings of this variability, and the learning it supports, are poorly understood.<br><br>Findings:<br>To establish a platform for the molecular analysis of song variability and plasticity, we have generated an initial draft assembly of the Bengalese finch genome from a single male animal to 151x coverage and an N50 of 3.0 MB. Furthermore, we have developed an initial set of gene models using RNA-seq data from eight samples that comprise liver, muscle, cerebellum, brainstem/midbrain and forebrain tissue from juvenile and adult Bengalese finches of both sexes.<br><br>Conclusions:<br>We provide a draft Bengalese finch genome and gene annotation to facilitate the study of the molecular-genetic influences on behavioral variability and the process of vocal learning. These data will directly support many avenues for the identification of genes involved in learning, including differential expression analysis, comparative genomic analysis (through comparison to extant avian genomes), and derivation of genetic maps for linkage analysis. Bengalese finch gene models and sequences will be essential for subsequent manipulation (molecular or genetic) of genes and gene products, enabling novel mechanistic investigations into the role of variability in learned behavior.
**Corresponding Author:**	Bradley Mark Colquitt, Ph.D.<br>University of California San Francisco<br>San Francisco, CA UNITED STATES
**Corresponding Author Secondary Information:**	
**Corresponding Author's Institution:**	University of California San Francisco
**Corresponding Author's Secondary Institution:**	
**First Author:**	Bradley Mark Colquitt, Ph.D.
**First Author Secondary Information:**	
**Order of Authors:**	Bradley Mark Colquitt, Ph.D.<br>David G Mets, Ph.D.<br>Michael Scott Brainard, Ph.D.
**Order of Authors Secondary Information:**	

Powered by Editorial Manager® and ProduXion Manager® from Aries Systems Corporation

Response to Reviewers:	We would like to thank the reviewers for their review of the manuscript and their helpful comments. We believe we have addressed the reviewers' specific concerns through several changes to the manuscript, as indicated below. In particular, each reviewer wished to see more detail concerning the manual curation of the gene annotation, and we have expanded this portion of the text:<br><br>Lines 200-209: "All models were then manually curated as follows using Apollo v2.0.4 [37]. Where possible, we corrected MAKER models that merged two genes, incorrectly split genes, or contained non-canonical splice junctions to eliminate frame shifts or truncated open reading frames and to best match aligned protein sequences. 3' UTR positions were manually refined by selecting from the longest 3' UTR in the Cufflinks assembled transcripts without allowing overlaps between UTRs and adjacent genes on the same strand. These criteria were used to better facilitate read-gene assignment in 3' RNA-sequencing experiments. The most well-represented 5' UTRs were selected from the Cufflinks assembled transcripts. This curation yielded a set of 15,322 genes (the increase in gene number occurred due to splitting of some incorrectly merged genes and inclusion of well-supported genes from the Cufflinks transcript models that had been excluded by MAKER)."<br><br>Reviewer #1: This is a simple, nicely written manuscript that presents the first assembly and annotation of the Bengalese finch genome. The rationale for the work is clearly described, and the methodological approach appears solid. My only request is for addition of few bits of information which may be helpful to others wishing to use the resource, specifically:<br><br>1 - details of the options used with Trimgalore, STAR, and Cufflinks v17<br>This is now described in the revised manuscript.<br><br>Lines 168-171: "TruSeq adaptor trimming was performed using: trim_galore --quality 20 -a AGATCGGAAGAG -a2 AGATCGGAAGAG --stringency 1. Nextera adaptor trimming was performed using: trim_galore --quality 20 -a CTGTCTCTTATA -a2 CTGTCTCTTATA --stringency 1."<br><br>Lines 182-184: "STAR v2.4.0h [33] set to remove non-canonical intron motifs (--outSAMstrandField intronMotif --outSAMattributes NH HI AS nM XS --outFilterIntronMotifs RemoveNoncanonical, otherwise default parameters)"<br><br>Lines 185-186: "...then assembled into transcripts using Cufflinks v2.2.1 [34] (-j .5 –min-frags-per-transfrag 50 –max-intron-length 1000000, otherwise default parameters)."<br><br>2 - how were the initial gene models derived for training Augustus, and how many were used?<br>This is now described in the revised manuscript.<br><br>Lines 196-197: "A random subset of gene models from the first MAKER2 run (n=3859) was used to train Augustus v2.5.5"<br><br>3- were all 15,313 final gene models manually curated in Apollo, or only some?<br>All models were manually curated.<br><br>4 - what criteria were employed for extending 3' UTRs?<br>The revised manuscript includes a fuller description of the criteria used for manual curation. Please see above.<br><br><br>Reviewer #2: The study of songbirds has contributed critically to our understanding of the neural basis for learning, skilled motor behavior, sexual differentiation of the brain, and countless other topics. The recent availability of multiple high-quality avian genome assemblies has provided a starting point to explore complex questions about the genetic basis and evolution of behavior. The authors provide a high-quality genome assembly for the Bengalese finch, including a set of curated gene annotations and transcriptome data from multiple tissues. This provides a much-needed resource to the many researchers interested in this important model organism. I strongly recommend

Powered by Editorial Manager® and ProduXion Manager® from Aries Systems Corporation

the paper for submission, following some minor revisions, which I have listed below by line number.

91 - 93 - Please split this run-on sentence into two separate sentences.
Thank you for identifying this error. We have corrected it.

112 - "high coverage," should be high-coverage.
We have made this change in the revised manuscript.

120 - "low coverage," should be low-coverage.
We have made this change in the revised manuscript.

197 - Here the authors state that they manually curated models to ensure completeness and to refine UTR positions. Please provide some brief description as to the logic used to guide the curatorial process. Did the authors use aligned Cufflinks RNA-seq transcripts to guide curation, or aligned models from other species, both, neither, or something else? If it is possible to determine at this point, approximately how many gene models required manual curation?
All genes were curated. The revised manuscript has been modified to indicate this. The revised manuscript also includes a fuller description of the criteria used for manual curation. Please see above.

199 - The authors state that BLASTP was used to align ORFs to the Uniprot-SwissProt protein database. Please specify the parameters used for the BLAST alignment, default or otherwise.
This is now described in the revised manuscript.

Lines 211-212: "...using BLASTP [38] (default parameters except -max_target_seqs 1)"

208 - Here the authors reference Figure 3, which provides a comparison of the Bengalese finch assembly and annotation with the assemblies from the Avian Phylogenomics Project. This figure was helpful for me to get a sense of how this assembly stacks up against previously available avian genomes. I think it would benefit the reader for the authors to provide in text some qualitative summary of the figure.
A description of how this assembly compares with those in the Avian Phylogenomics project is now provided at the end of 'Data Description'.

Lines 109-111: "This assembly has coverage and scaffolding length that are on the upper ends of the distribution of assemblies in the Avian Phylogenomics project [26] and has a comparable number of gene models (Fig. 2)."

Table 1:
- Please specify the units for the Age column (e.g. post-hatch days).
- RNA libraries: the table should be alphabetized by tissue type.
- RNA libraries: one of the rows is labeled "Midbrain/brain" where it should be labeled "Midbrain/brainstem."
- RNA libraries: the NCBI records for the RNA-seq samples indicate that the muscle sample is specifically breast muscle. There's no reason not to specify "Breast muscle" in this table also.
We have made these changes to Table 1 in the revised manuscript.

Reviewer #3: Comments to manuscript GIGA-D-17-00224.

General:
The manuscript by Colquitt et al. is a short technical note presenting the development of a draft genome assembly and a set of RNAseq data sets for the Bengalese finch. Besides the assembly stats comparison to previously published avian genomes in the frame-work of the avian genomics consortium, the manuscript contains no analyses. Hence, this review only considers the rationale behind selecting this particular species and the technical aspects of generating the data. The manuscript is well written and easy to follow.

The authors state that the Bangalese finch could develop into a model system for understanding the genetic basis of vocal learning, in particular of song variability and

Powered by Editorial Manager® and ProduXion Manager® from Aries Systems Corporation

	plasticity. In the introduction, the authors make a thorough review of the status of the Bengalese finch in the field of vocal learning. Admittedly, I was initially a bit surprised that the manuscript contained such a detailed introduction describing the study organism but after several rounds of reading I conclude that this strengthens the paper and makes the rationale behind developing genomic tools for this species well supported.<br><br>The methods section clearly states how the data was collected and edited before submission to data bases. I could access the data via the links provided so it should be accessible for researchers interested in using these data for analyses. The data was curated with standard methods in the field. My only concerns regard, i) the manual curation of the gene models which could be described in more detail (did you omit ORF:s out of frame?, change the sequence to get ORF in frame?, how was UTR positions determined?, etc.), and, ii) a better description of the rationale behind selecting the particular tissues/sexes for RNAseq.<br><br>Detailed comments:<br>L71 omit 'the'<br>Thank you for identifying this error. We have corrected it.<br><br>L83 Is this statement correct? Several songbirds have very high-quality assemblies available (eg. Ficedula albicollis, Corvus corone).<br>The reviewer is correct that this statement is no longer accurate. By scaffold N50, the canary (Serinus canaria) and hooded crow (Corvus cornix cornix) both have better assembled genomes. The sentence no longer has this assertion and now reads:<br><br>Lines 82-84: “The zebra finch (Taenopygia guttata), another commonly used model for vocal learning, shared a most recent common ancestor with the white-rumped munia ~9 MYA.”<br><br>L100 It is stated that the Bengalese finch has high level of genetic polymorphism - in the range of outbred human populations. Most songbirds have considerably higher polymorphism levels than that. I would suggest to present this in a different way and give the estimated theta values.<br><br>As is suggested by the reviewer, to avoid confusion on this point, a significant elaboration would be required to clearly discuss the details of our measure of nucleotide diversity. Such an elaboration would be beyond the scope of this document and so, we have removed the statement about genetic diversity.<br><br>L130 Maybe provide approved animal use protocol (ID number).<br>Line 128: We have made this change in the revised manuscript.<br><br>L182 Ref for TrimGalore!<br>Line 168: We have made this change in the revised manuscript.<br><br>L190-191. Please, provide dates for accession/download since these data bases sometimes change.<br>We have made this change in the revised manuscript.<br><br>Line 194: “Zebra finch EST collection (taeGut2) downloaded from UCSC (on Jan 11, 2015).”<br><br>L202-203. As far as I am aware CEGMA is not recommended anymore after the BUSCO tool was developed. The CEGMA part can hence be omitted. Unclear here also how 65% can be complete and 94% partial CEGs?<br>We have removed the CEGMA analysis.<br><br>L332 typos; of, shown?<br>Line 323: Thank you for identifying this error. We have corrected it.
**Additional Information:**	
**Question**	**Response**

Powered by Editorial Manager® and ProduXion Manager® from Aries Systems Corporation

Are you submitting this manuscript to a special series or article collection?	No
**Experimental design and statistics**<br><br>Full details of the experimental design and statistical methods used should be given in the Methods section, as detailed in our Minimum Standards Reporting Checklist. Information essential to interpreting the data presented should be made available in the figure legends.<br><br>Have you included all the information requested in your manuscript?	Yes
**Resources**<br><br>A description of all resources used, including antibodies, cell lines, animals and software tools, with enough information to allow them to be uniquely identified, should be included in the Methods section. Authors are strongly encouraged to cite Research Resource Identifiers (RRIDs) for antibodies, model organisms and tools, where possible.<br><br>Have you included the information requested as detailed in our Minimum Standards Reporting Checklist?	Yes
**Availability of data and materials**<br><br>All datasets and code on which the conclusions of the paper rely must be either included in your submission or deposited in publicly available repositories (where available and ethically appropriate), referencing such data using a unique identifier in the references and in the "Availability of Data and Materials" section of your manuscript.<br><br>Have you have met the above requirement as detailed in our Minimum Standards Reporting Checklist?	Yes

Manuscript Click here to download Manuscript BengaleseFinch-woTables6-2-18.odt

Draft genome assembly of the Bengalese finch, *Lonchura striata domestica,* a model for motor skill variability and learning

Bradley M. Colquitt[1,*] David G. Mets[1,] Michael S. Brainard[1,2]

1. Department of Physiology, University of California-San Francisco, San Francisco, California, USA
2. Howard Hughes Medical Institute, Chevy Chase, Maryland, USA

* Corresponding author: bradley.colquitt@ucsf.edu

Abstract

Background:

Vocal learning in songbirds has emerged as a powerful model for sensorimotor learning. Neuro-behavioral studies of Bengalese finch (*Lonchura striata domestica*) song, naturally more variable and plastic than songs of other finch species, have demonstrated the importance of behavioral variability for initial learning, maintenance, and plasticity of vocalizations. However, the molecular and genetic underpinnings of this variability, and the learning it supports, are poorly understood.

Findings:

To establish a platform for the molecular analysis of behavioral variability and plasticity, we have generated an initial draft assembly of the Bengalese finch genome from a single male animal to 151x coverage and an N50 of 3.0 MB. Furthermore, we have developed an initial set of gene models using RNA-seq data from eight samples that comprise liver, muscle, cerebellum, brainstem/midbrain and forebrain tissue from juvenile and adult Bengalese finches of both sexes.

Conclusions:

We provide a draft Bengalese finch genome and gene annotation to facilitate the study of the molecular-genetic influences on behavioral variability and the process of vocal learning. These data will directly support many avenues for the identification of genes involved in learning, including differential expression analysis, comparative genomic analysis (through comparison to existing avian genome assemblies), and derivation of genetic maps for linkage analysis. Bengalese finch gene models and sequences will be essential for subsequent manipulation (molecular or genetic) of genes and gene products, enabling novel mechanistic investigations into the role of variability in learned behavior.

Keywords

Genome assembly, systems neuroscience, molecular neuroscience, neural plasticity, birdsong, Bengalese finch

Data Description

Many motor skills, from walking and talking to the swing of a baseball bat, have the capacity for high degrees of both stability and flexibility between renditions. This capacity allows organisms to both reliably perform well-learned behaviors and to adapt behaviors in settings that present new environmental information. Regulation of this balance is a fundamental aspect of neural function, and its disruption may underlie neurological diseases characterized by excessive motor rigidity or variability, such as Parkinson's and Huntington's diseases [1,2]. Hence, understanding the neural mechanisms that mediate maintenance and adaptive modification of motor skills is critical to understanding the basis of both normal and pathological behavior.

The songs of songbirds are complex vocal motor skills and provide a powerful framework through which to understand the neural mechanisms that regulate motor skill learning, maintenance, and plasticity [3–5]. As with motor skills in humans, birdsong is learned and must be practiced to maintain performance.

In particular, birdsong learning follows a similar developmental trajectory to human speech learning: song is initially acquired during an early critical period followed by a period of practice and then relatively invariant song production throughout adulthood [6]. Adult song relies on auditory feedback both to maintain song at a stable setpoint and to support adaptive change in response to environmental perturbations. Importantly, song production and learning is subserved by an anatomically discrete and functionally dedicated set of brain nuclei, which allows targeted characterization of electrophysiological and molecular properties of those nuclei that can be related back to song production, learning, and plasticity.

Relative to the songs of other commonly studied songbirds, the song of the Bengalese finch has several experimentally useful features that facilitate the study of behavioral variability in both learning and maintenance of complex behaviors. Bengalese finches (Fig. 1) exhibit substantial rendition-to-rendition variability in both the ordering and phonological attributes of their song elements [7]. This natural variation acts as a substrate for error-corrective and reinforcement learning [8–12] and has facilitated the analysis of how fluctuations in central nervous system activity lead to behavioral variation [13–15]. Furthermore, Bengalese finch song is more sensitive to auditory feedback and operant training paradigms than the songs of other songbird species. Complete loss of auditory feedback results in an increase in song sequence variability and rapid degradation of its spectral content [16,17]. Experiments using subtler distortions of auditory feedback indicate that Bengalese finches make corrections to adaptively adjust their song to minimize errors [9,18]. These studies, facilitated by behavior specific to the Bengalese finch, have provided insight into the neural mechanisms driving variability and how that variability facilitates learning. However, studies of the molecular mechanisms which support this variability have been precluded by the absence of a genome assembly.

Beyond facilitating molecular studies of learning, this genome assembly is the first of a species in the genus *Lonchura,* which comprises approximately 37 species variously called munias or mannikins.

Recent constructions of the Estrildid clade indicate that the *Lonchura* genus is monophyletic (with the exceptions of the African (*L. cantans*) and Indian (*L. malabarica*) silverbills) and radiated approximately 6 million years ago (MYA) [19–21]. The zebra finch (*Taenopygia guttata*), another commonly used model for vocal learning, shared a most recent common ancestor with the white-rumped munia ~9 MYA. The assembly provided here presents an opportunity for further comparative genomic work as well as molecular genetic analysis in a previously poorly studied genus.

Bengalese finches are a domesticated variant of the white-rumped munia (*Lonchura striata),* an Estrildid finch that is indigenous to Southeast Asia including India, Myanmar, Thailand, Malaysia, and South China [22]. The birds are socially gregarious and live in large colonies that forage through open grasslands and urban backyards. The first well-documented case of domestication of the white-rumped munia is thought to have occurred approximately 250 years ago at the request of a Japanese feudal lord. Since then, the species has been selectively bred for tameness and reproductive efficiency [23]. Today, Bengalese finches (also known as Society finches) are widely kept as household pets. Interestingly, although there is no clear evidence that the Bengalese finch was bred for certain song characteristics, comparisons of the songs of the ancestral white-rumped munia and the Bengalese finch indicate that domestication has resulted in increased song complexity and a broader capacity to learn the songs of both the wild and domesticated variants [24,25]. Domestication has also led to laboratory populations that exhibit substantial interindividual variation in both plumage and song characteristics. The addition of a genome sequence for a domesticated species opens opportunities for comparative analysis into the impact of domestication on the genome.

Several songbird genome assemblies have been generated in recent years, including genomes for the zebra finch [26], canary [27], and American crow [28], opening up songbirds to genome-wide molecular analysis. However, the unique song features of Bengalese finches provide a system ideally suited to

address specific questions regarding the molecular properties of the song system that facilitate or constrain song variability and the ability to respond to altered environmental conditions.

To lay the groundwork for molecular studies in the Bengalese finch, we generated a high-coverage draft genome assembly and constructed an initial set of gene annotations. This assembly has coverage and scaffolding length that are on the upper ends of the distribution of assemblies in the Avian Phylogenomics project [28] and has a comparable number of gene models (Fig. 2).

Re-use potential

We expect that this resource will be used by other researchers for differential expression analysis, functional genomics, and comparative genomic analysis (through comparison to existing avian genomes), with a specific application to characterizing the differences between the genomes of the Bengalese finch and its ancestral species that contribute to differences in their songs [23]. The assembly can also be used as a reference for low-coverage sequencing and marker typing experiments examining how genetic variation within a laboratory population contributes to heritable variation in song. Additionally, these gene models and sequences will be essential for manipulation (molecular or genetic) of genes and gene products, a prerequisite for developing models for molecular mechanisms. Moreover, this is the first large-scale genome assembly of a member of the *Lonchura* genus and will aid in further reconstructions of Estrildid phylogeny and in songbird evolution generally.

Methods

Animals

All birds were raised in our breeding colony at UCSF, and experiments were conducted in accordance with NIH and UCSF policies governing animal use and welfare (protocol number AN170723-01A).

Genomic DNA library construction

Blood was collected from a single Bengalese finch adult male and purified using DNeasy Blood & Tissue Kit (Qiagen).

We prepared two sets of libraries for genome assembly: one set with small insert size libraries and a second with larger insert size mate-pair libraries. First, small insert size libraries with two different sizes were constructed. Two samples of 2.2 ug of genomic DNA were sonicated using a Covaris M220, 130 µl microTUBE, and presets for a target size of 200 bp (peak incident power 50 W, duty factor 20%, cycles per burst 200, treatment time 160 s). Samples were then purified using Sample Purification Beads (Illumina). Libraries were prepared from this sonicated gDNA using the TruSeq DNA PCR-Free LT Library Preparation Kit (Illumina). Briefly, samples were end repaired using End Repair Mix 2 then bead purified. Samples were then size selected using a BluePippin 2% agarose, dye-free, external marker gel (Sage Biosciences) set for 200 and 220 bp tight selection. Samples were then a-tailed, adapter ligated, and purified as indicated in the manufacturer's protocol.

Next, mate-pair libraries were constructed using the Nextera Mate-Pair Library Preparation Kit (Illumina) with 3, 5, and 9 kb insert sizes. 4 µg purified genomic DNA was tagmented as recommended in the manufacturer's protocol then purified using the Genomic DNA Clean and Concentrator Kit (Zymo). The protocol was continued through strand displacement and size selected using BluePippin 0.75% agarose, dye-free gels (broad selection at 2000-4000 bp, 4000-6000 bp, and 8000-10,000 bp respectively). After selection, the protocol was continued through final PCR amplification.

RNA collection and library construction

All tissues were dissected out then minced and homogenized on ice. RNA was extracted using standard TRIzol extraction. 2 µg total RNA was DNase-treated using 2U rDNase I (Ambion) at 37°C for 25 minutes. DNase-treated total RNA was purified using RNA Clean and Concentrator 25 (Zymo) then 120 ng of this sample was prepared for sequencing using the Encore Complete DR RNA-seq Library System

(NuGEN) according to the manufacturer's protocol. Table 1 provides tissue information including sex and ages of the animals.

Sequencing

Small insert, mate-pair, and total RNA libraries were sequenced on eight lanes of an Illumina HiSeq 2500 using V4 chemistry at Elim Biopharm (Hayward, CA). Libraries were sequenced paired end to 125 cycles. Sequencing statistics are found in Table 1.

Genome assembly

Sequencing data was assembled at the UC Davis Genome Center using ALLPATHS-LG (ALLPATHS-LG, RRID:SCR_010742) [29]. Prior to assembly, reads were trimmed for TruSeq (fragment libraries) or TruSeq and Nextera (jumping libraries) adapters using Trim Galore! [30], a wrapper for CutAdapt [31] and FastQC (FastQC, RRID:SCR_014583) [32]. TruSeq adaptor trimming was performed using: trim_galore --quality 20 -a AGATCGGAAGAG -a2 AGATCGGAAGAG --stringency 1. Nextera adaptor trimming was performed using: trim_galore --quality 20 -a CTGTCTCTTATA -a2 CTGTCTCTTATA --stringency 1. ALLPATHS-LG was then run using standard parameters. Statistics for the resulting assembly are in Table 2.

Repeat masking

The genome assembly was first masked for simple repeats and using specific repeat models generated using RepeatMasker open-4.0.5 [33] with -lib flag set using custom families generated using RepeatModeler open-1.0.8 [34]. Approximately 7.5% of the genome was classified as repetitive, comprising 80 Mbase of DNA. More detailed repeat element statistics can be found in Table 3.

Transcript assembly and gene annotation

RNA library sequencing reads were first trimmed for TruSeq adapters using Trim Galore! (as above). Reads were aligned to the genome assembly using STAR v2.4.0h [35] set to remove non-canonical intron motifs (--outSAMstrandField intronMotif --outSAMattributes NH HI AS nM XS --outFilterIntronMotifs RemoveNoncanonical, otherwise default parameters), then assembled into transcripts using Cufflinks v2.2.1 (Cufflinks, RRID:SCR_014597) [36] (-j .5 –min-frags-per-transfrag 50 –max-intron-length 1000000, otherwise default parameters).

Gene annotation was performed using the MAKER2 pipeline [37] (Fig. 3). The following sources of evidence were used:

1) Cufflinks transcript assembly described above
2) A collection of UniProt protein sequences from human, mouse, chicken, and zebra finch (each downloaded March 2, 2017).
3) Zebra finch EST collection (taeGut2) downloaded from UCSC (on Jan 11, 2015).

A random subset of gene models from the first MAKER2 run (n=3859) was used to train Augustus v2.5.5 (Augustus: Gene Prediction, RRID:SCR_008417) [38], and the MAKER2 pipeline was re-run using these models to improve annotation. 3' UTRs were added by intersecting these gene models with Cufflinks generated transcripts. MAKER2 generated 17,268 gene models that were filtered by AED scores below 0.5 (a measure of model support) to yield 15,313 models. All models were then manually curated as follows using Apollo v2.0.4 (Apollo, RRID:SCR_001936) [37]. Where possible, we corrected MAKER models that merged two genes, incorrectly split genes, or contained non-canonical splice junctions to eliminate frame shifts or truncated open reading frames and to best match aligned protein sequences. 3' UTR positions were manually refined by selecting from the longest 3' UTR in the Cufflinks assembled transcripts without allowing overlaps between UTRs and adjacent genes on the same strand. These criteria were used to better facilitate read-gene assignment in 3' RNA-sequencing experiments.

The most well-represented 5' UTRs were selected from the Cufflinks assembled transcripts. This curation yielded a set of 15,322 genes (the increase in gene number occurred due to splitting of some incorrectly merged genes and inclusion of well-supported genes from the Cufflinks transcript models that had been excluded by MAKER). Open reading frame sequences were aligned to the Uniprot-SwissProt protein database (downloaded 3/20/2015) using BLASTP [40] (default parameters except -max_target_seqs 1), which yielded 14,449 genes with a protein assignment with e-value less than 10^{-10}.

BUSCO (BUSCO, RRID:SCR_015008) [41], which detects near-universal single-copy orthologs to assay genome completeness, yielded 86% complete (n=2621), 4% fragmented (n=122), and 9% missing (n=280) vertebrate genes (total n=3023).

A comparison of this assembly and annotation with the assemblies in the Avian Phylogenomics Project can be found in Figure 2. The full assembly and annotation were submitted to NCBI using custom scripts, GAG [42], Annie [43], and NCBI tbl2asn.

The authors declare that they have no competing interests.

Availability of data

This Whole Genome Shotgun project has been deposited at DDBJ/ENA/GenBank under the accession MUZQ00000000. The version described in this paper is version MUZQ01000000. Supporting data, including transcriptome data, annotations, BUSCO results and scripts are available via the *GigaScience* repository GigaDB [44].

Funding

This work was supported by the National Institute of Neurological Disorders and Stroke (F32NS098809) and the Howard Hughes Medical Institute.

Authors' contributions

BC designed the project, performed all experiments and analysis, and wrote the manuscript. DM and MB conceived and designed the project.

Acknowledgements

We thank Dr. Joe Fass and Richard Feltstykeet from the UC Davis Genome Center Bioinformatics Core for their tremendous help and consultation, which contributed to the success of this project. We also thank Foad Green for his help manually curating the gene annotation.

References

1. Wolpert DM, Diedrichsen J, Flanagan JR. Principles of sensorimotor learning. Nat. Rev. Neurosci. 2011;12:739–51.

2. Doyon J. Motor sequence learning and movement disorders. Curr. Opin. Neurol. 2008;21:478–83.

3. Brainard MS, Doupe AJ. What songbirds teach us about learning. Nature. 2002;417:351–8.

4. Brainard MS, Doupe AJ. Translating birdsong: songbirds as a model for basic and applied medical research. Annu. Rev. Neurosci. 2013;36:489–517.

5. Konishi M. Birdsong for neurobiologists. Neuron. 1989;3:541–9.

6. Doupe AJ, Kuhl PK. Birdsong and human speech: common themes and mechanisms. Annu. Rev. Neurosci. 1999;22:567–631.

7. Okanoya K. The Bengalese Finch: A Window on the Behavioral Neurobiology of Birdsong Syntax. Ann. N. Y. Acad. Sci. 2004;1016:724–35.

8. Tumer EC, Brainard MS. Performance variability enables adaptive plasticity of "crystallized" adult birdsong. Nature. 2007;450:1240–4.

9. Sober SJ, Brainard MS. Adult birdsong is actively maintained by error correction. Nat. Neurosci. 2009;12:927–31.

10. Warren TL, Tumer EC, Charlesworth JD, Brainard MS. Mechanisms and time course of vocal learning and consolidation in the adult songbird. J. Neurophysiol. 2011;106:1806–21.

11. Warren TL, Charlesworth JD, Tumer EC, Brainard MS. Variable sequencing is actively maintained in a well learned motor skill. J. Neurosci. 2012;32:15414–25.

12. Charlesworth JD, Tumer EC, Warren TL, Brainard MS. Learning the microstructure of successful behavior. Nat. Rev. Neurosci. 2011;14:373–80.

13. Sober SJ, Wohlgemuth MJ, Brainard MS. Central contributions to acoustic variation in birdsong. J. Neurosci. 2008;28:10370–9.

14. Fujimoto H, Hasegawa T, Watanabe D. Neural Coding of Syntactic Structure in Learned Vocalizations in the Songbird. J. Neurosci. 2011;31.

15. Wohlgemuth MJ, Sober SJ, Brainard MS. Linked control of syllable sequence and phonology in birdsong. J. Neurosci. 2010;30:12936–49.

16. Okanoya K, Yamaguchi A. Adult Bengalese finches (Lonchura striata var. domestica) require real-time auditory feedback to produce normal song syntax. J. Neurobiol. 1997;33:343–56.

17. Woolley SM, Rubel EW. Bengalese finches Lonchura Striata domestica depend upon auditory feedback for the maintenance of adult song. J. Neurosci. 1997;17:6380–90.

18. Sakata JT, Brainard MS. Real-time contributions of auditory feedback to avian vocal motor control. J. Neurosci. Society for Neuroscience; 2006;26:9619–28.

19. Hooper DM, Price TD. Rates of karyotypic evolution in Estrildid finches differ between island and continental clades. Evolution (N. Y). 2015;69:890–903.

20. Arnaiz-Villena A, Ruiz-Del-Valle V, Gomez-Prieto P, Reguera R, Parga-Lozano C, Serrano-Vela I. Estrildinae Finches (Aves, Passeriformes) from Africa, South Asia and Australia: a Molecular Phylogeographic Study. Open Ornithol. J. 2009;2:29–36.

21. Sorenson MD, Balakrishnan CN, Payne RB, Johnson K. Clade-Limited Colonization in Brood Parasitic Finches (Vidua spp.). Syst. Biol. Sinauer, Sunderland, Massachusetts; 2004;53:140–53.

22. Restall R. Munias and Mannikins. East Sussex, UK: Pica Press; 1996.

23. Okanoya K. Evolution of song complexity in Bengalese finches could mirror the emergence of human language. J. Ornithol. 2015;156:65–72.

24. Honda E, Okanoya K. Acoustical and Syntactical Comparisons between Songs of the White-backed Munia (Lonchura striata) and Its Domesticated Strain, the Bengalese Finch (Lonchura striata var. domestica). Zoolog. Sci. 1999;16:319–26.

25. Takahasi M, Okanoya K. Song Learning in Wild and Domesticated Strains of White-Rumped Munia, Lonchura striata , Compared by Cross-Fostering Procedures: Domestication Increases Song Variability by Decreasing Strain-Specific Bias. Ethology. 2010;116:396–405.

26. Warren WC, Clayton DF, Ellegren H, Arnold AP, Hillier LW, Künstner A, et al. The genome of a songbird. Nature. 2010;464:757–62.

27. Frankl-Vilches C, Kuhl H, Werber M, Klages S, Kerick M, Bakker A, et al. Using the canary genome to decipher the evolution of hormone-sensitive gene regulation in seasonal singing birds. Genome Biol. 2015;16:19.

28. Zhang G, Li B, Li C, Gilbert MTP, Jarvis ED, Wang J. Comparative genomic data of the Avian Phylogenomics Project. Gigascience. 2014;3:26.

29. Gnerre S, Maccallum I, Przybylski D, Ribeiro FJ, Burton JN, Walker BJ, et al. High-quality draft assemblies of mammalian genomes from massively parallel sequence data. Proc. Natl. Acad. Sci. U. S. A. 2011;108:1513–8.

30. Krueger F. Trim Galore! [Internet]. 2014. Available from: https://github.com/FelixKrueger/TrimGalore

31. Martin M. Cutadapt removes adapter sequences from high-throughput sequencing reads. EMBnet.journal. 2011;17:10.

32. Andrews S. FastQC. 2015.

33. Smit A, Hubley R, Green P. RepeatMasker Open-4.0. 2013.

34. Smit AFA, Hubley R. RepeatModeler Open-1.0. 2010.

35. Dobin A, Davis CA, Schlesinger F, Drenkow J, Zaleski C, Jha S, et al. STAR: ultrafast universal RNA-seq aligner. Bioinformatics. 2013;29:15–21.

36. Roberts A, Pimentel H, Trapnell C, Pachter L. Identification of novel transcripts in annotated genomes using RNA-Seq. Bioinformatics. 2011;27:2325–9.

37. Holt C, Yandell M. MAKER2: an annotation pipeline and genome- database management tool for second- generation genome projects. BMC Bioinformatics. 2011;12.

38. Stanke M, Keller O, Gunduz I, Hayes A, Waack S, Morgenstern B. AUGUSTUS: ab initio prediction of alternative transcripts. Nucleic Acids Res. 2006;34:W435-9.

39. Lee E, Helt GA, Reese JT, Munoz-Torres MC, Childers CP, Buels RM, et al. Web Apollo: a web-based genomic annotation editing platform. Genome Biol. 2013;14.

40. Camacho C, Coulouris G, Avagyan V, Ma N, Papadopoulos J, Bealer K, et al. BLAST+: architecture and applications. BMC Bioinformatics. 2009;10:421.

41. Simão FA, Waterhouse RM, Ioannidis P, Kriventseva E V., Zdobnov EM, S.C. C, et al. BUSCO: assessing genome assembly and annotation completeness with single-copy orthologs. Bioinformatics. 2015;31:3210–2.

42. Hall B, DeRego T, Geib S. GAG: the Genome Annotation Generator (Version 1.0). 2014.

43. Tate R, Hall B, DeRego T, Geib S. Annie: the ANNotation Information Extractor (Version 1.0). 2014.

44. Colquitt BM, Mets DG, Brainard MS. Supporting data for “Draft genome assembly of the Bengalese finch, Lonchura striata domestica, a model for motor skillvariability and learning”. *GigaScience* Database 2018. http://dx.doi.org/10.5524/100398

Figure legends

Figure 1. Bengalese finch (*Lonchura striata domestica*). An adult male Bengalese finch.

Figure 2. Comparison of Bengalese finch and Avian Phylogenomics Project assemblies. The distributions of sequencing depths (A), scaffold N50 (B), and number of annotated genes (C) are shown for the assemblies in the Avian Phylogenomics Project as of September 14, 2017. Vertical red line indicates the corresponding statistics for the Bengalese finch assembly and annotation described here.

Figure 3. Flowchart of genome assembly and annotation. Experimental and computational approach used for genome assembly and gene annotation.

Table legends

Table 1. Descriptions of libraries used for genome assembly and gene annotation.

Table 2. Statistics of draft genome assembly.

Table 3. Repeat elements in the genome assembly identified by RepeatMasker

Click here to download Table table1.pdf

Table 1. Sequencing libraries

Genomic libraries

Library	Insert size (expected)	Insert size (measured)	Reads (M)	Sequence (Gbases)	Coverage (x)
Fragment 1	200	202	403	50	42
Fragment 2	220	226	412	51	43
Jumping 1	3000	3300	753	60	50
Jumping 2	5000	5300	149	12	10
Jumping 3	9000	9000	100	7	6
Totals			1817	180	151

RNA libraries

Tissue	Sex	Age (days post hatch)	Reads (M)	Sequence (Gbases)
Cerebellum	male	360	153	19
Forebrain	female	194	179	22
Forebrain	male	147	159	20
Forebrain	female	55	266	33
Forebrain	male	55	160	20
Liver	female	217	148	18
Midbrain/brainstem	male	360	182	23
Breast muscle	female	217	193	24
Totals			1439	180

337 **Table 2. Assembly statistics**

**ALLPATHS-LG output**	
number of contigs	37187
number of contigs per Mb	35.1
number of scaffolds	3016
total contig length	1027319005
total scaffold length, with gap	1058688097
N50 scaffold size in kb, with gaps	2953
number of scaffolds per Mb	2.85
median size of gaps in scaffolds	270
% of bases in captured gaps	2.94

**Assemblathon statistics**	
Total scaffold length as percentage of assumed genome size	88.30%
% of estimated genome that is useful (>= 25 kb)	87.60%
Longest scaffold	15662897
Shortest scaffold	887
Number of scaffolds > 1K nt	2987 (99.0%)
Number of scaffolds > 10K nt	1254 (41.6%)
Number of scaffolds > 100K nt	719 (23.8%)
Number of scaffolds > 1M nt	297 (9.8%)
Number of scaffolds > 10M nt	3 (0.1%)
Mean scaffold size	351516
Median scaffold size	5349
N50 scaffold length	2953339
L50 scaffold count	103
NG50 scaffold length	2494006
LG50 scaffold count	129
N50 scaffold - NG50 scaffold length difference	459333
scaffold %A	28.31
scaffold %C	20.13
scaffold %G	20.09
scaffold %T	28.24
scaffold %N	2.94
Percentage of assembly in scaffolded contigs	99.60%
Percentage of assembly in unscaffolded contigs	0.40%
Average number of contigs per scaffold	10.5
Average length of break (>25 Ns) between contigs in scaffold	1082

Click here to download Table table3.pdf

338

Table 3. Repeat element statistics

Class	N	Total length (Mbases)	Percent of genome
DNA	3460	0.31	0.03
LINE	118051	32.03	3.03
Low_complexity	46755	2.66	0.25
LTR	66142	25.51	2.41
Satellite	3822	2.01	0.19
Simple_repeat	242428	11.94	1.13
SINE	2163	0.15	0.01
Unknown	14079	4.91	0.46
Total	496900	79.52	7.52

Figure 1. Bengalese finch (*Lonchura striata domestica)*

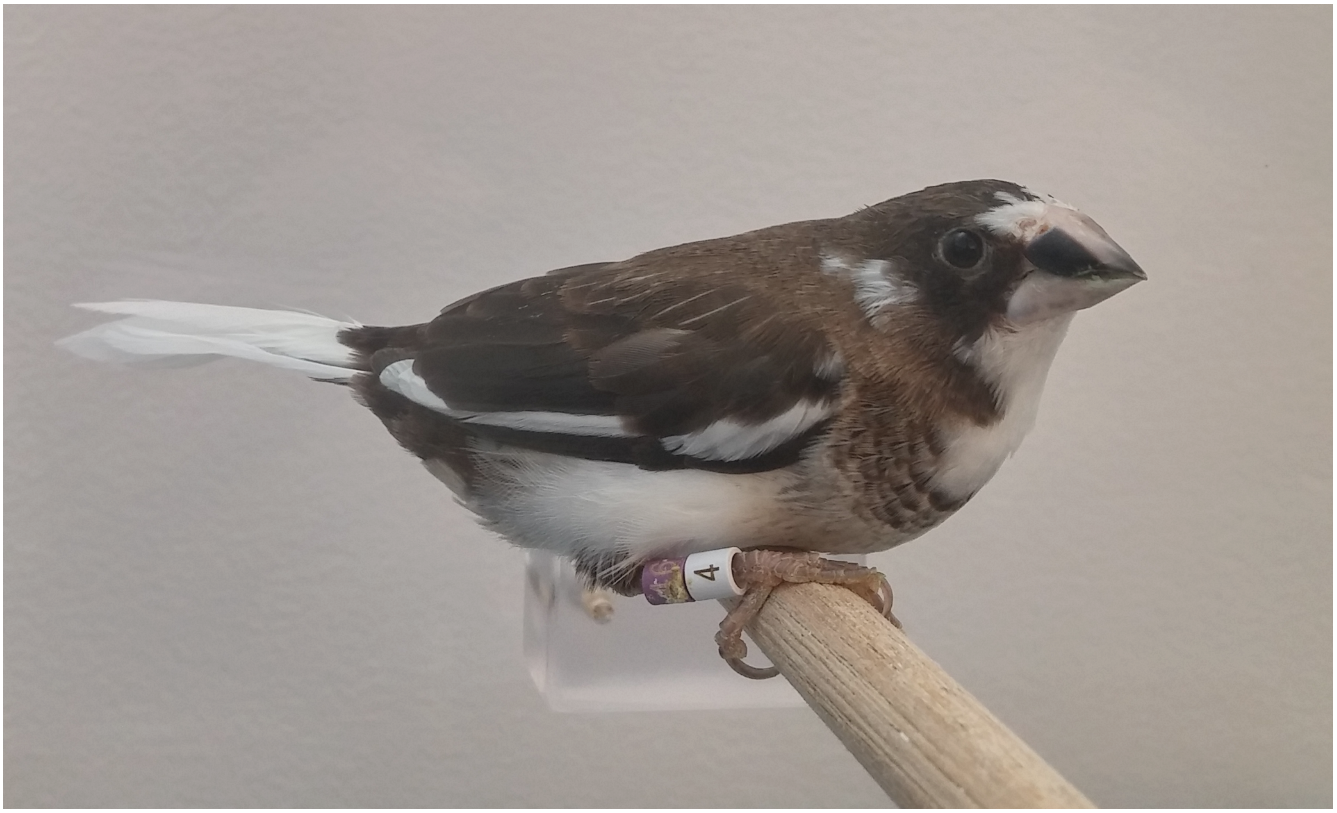

Figure 2

Click here to download Figure figure2.pdf

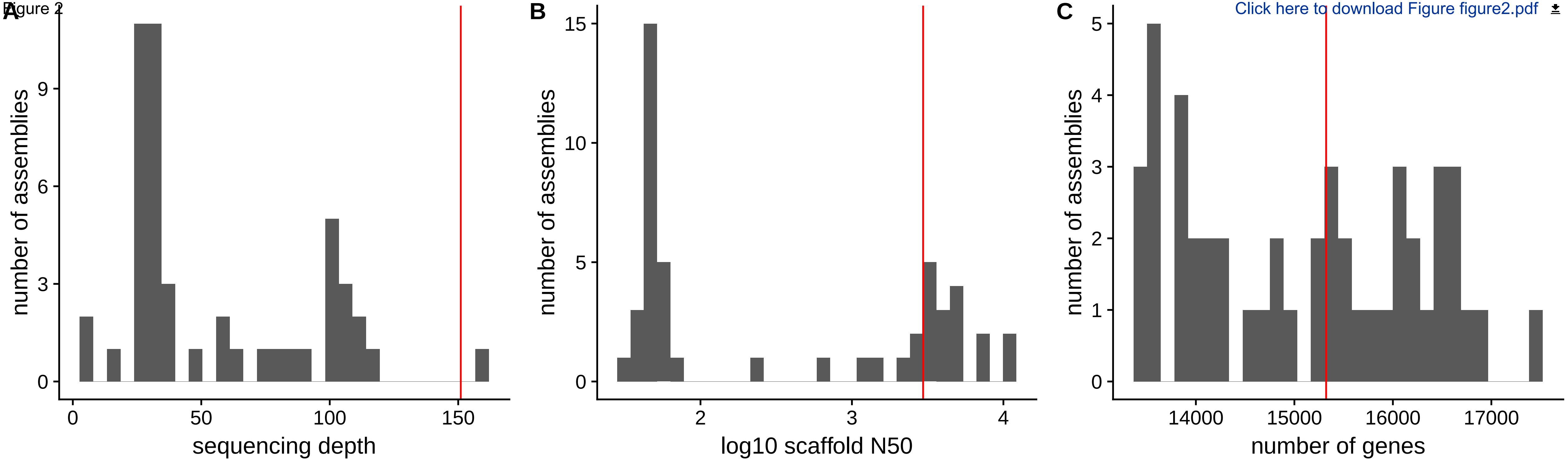

Click here to download Figure figure3.pdf

Figure 2. Flowchart of genome assembly and annotation.

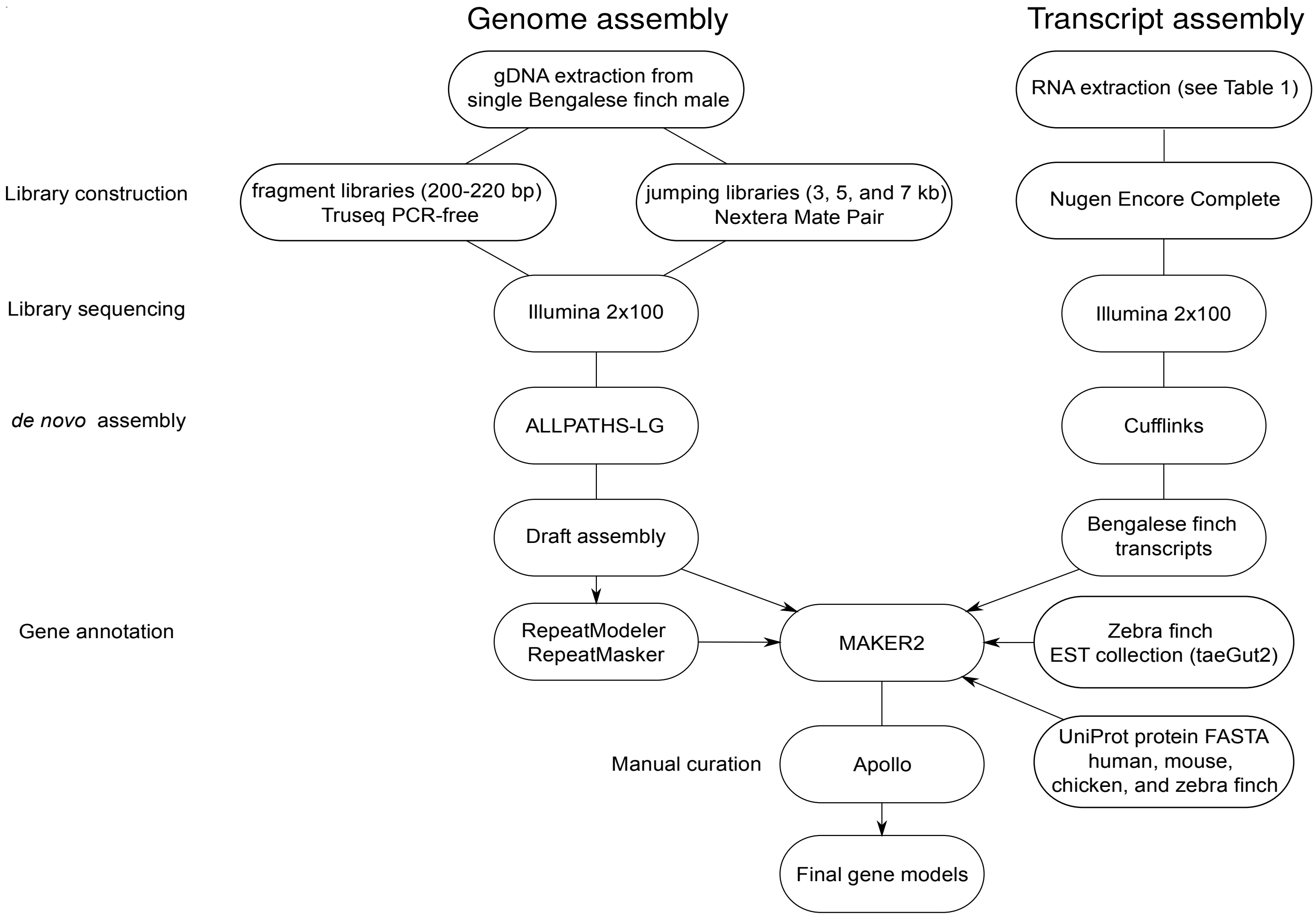

Supplement: GIGA-D-17-00224_Revision_1.pdf [file giy008_giga-d-17-00224_revision_1.pdf]
