# Supplementary material for: Draft genome assembly of the Bengalese finch, Lonchura striata domestica, a model for motor skill variability and learning
Source: Gigascience. 2018 Feb 15;7(3):giy008. doi: 10.1093/gigascience/giy008 (PMC5861438; doi:10.1093/gigascience/giy008)
Supplement: Response_to_Reviewer_Comments_Original_Submission.pdf [file giy008_response_to_reviewer_comments_original_submission.pdf]

Author's Response To Reviewer Comments

We would like to thank the reviewers for their review of the manuscript and their helpful comments. We believe we have addressed the reviewers' specific concerns through several changes to the manuscript, as indicated below. In particular, each reviewer wished to see more detail concerning the manual curation of the gene annotation, and we have expanded this portion of the text:

Lines 200-209: "All models were then manually curated as follows using Apollo v2.0.4 [37]. Where possible, we corrected MAKER models that merged two genes, incorrectly split genes, or contained non-canonical splice junctions to eliminate frame shifts or truncated open reading frames and to best match aligned protein sequences. 3' UTR positions were manually refined by selecting from the longest 3' UTR in the Cufflinks assembled transcripts without allowing overlaps between UTRs and adjacent genes on the same strand. These criteria were used to better facilitate read-gene assignment in 3' RNA-sequencing experiments. The most well-represented 5' UTRs were selected from the Cufflinks assembled transcripts. This curation yielded a set of 15,322 genes (the increase in gene number occurred due to splitting of some incorrectly merged genes and inclusion of well-supported genes from the Cufflinks transcript models that had been excluded by MAKER)."

Reviewer #1: This is a simple, nicely written manuscript that presents the first assembly and annotation of the Bengalese finch genome. The rationale for the work is clearly described, and the methodological approach appears solid. My only request is for addition of few bits of information which may be helpful to others wishing to use the resource, specifically:

1 - details of the options used with Trimgalore, STAR, and Cufflinks v17
This is now described in the revised manuscript.

Lines 168-171: "TruSeq adaptor trimming was performed using: trim_galore --quality 20 -a AGATCGGAAGAG -a2 AGATCGGAAGAG --stringency 1. Nextera adaptor trimming was performed using: trim_galore --quality 20 -a CTGTCTCTTATA -a2 CTGTCTCTTATA --stringency 1."

Lines 182-184: "STAR v2.4.0h [33] set to remove non-canonical intron motifs (--outSAMstrandField intronMotif --outSAMattributes NH HI AS nM XS --outFilterIntronMotifs RemoveNoncanonical, otherwise default parameters)"

Lines 185-186: "...then assembled into transcripts using Cufflinks v2.2.1 [34] (-j .5 –min-frags-per-transfrag 50 –max-intron-length 1000000, otherwise default parameters)."

2 - how were the initial gene models derived for training Augustus, and how many were used?
This is now described in the revised manuscript.

Lines 196-197: "A random subset of gene models from the first MAKER2 run (n=3859) was used to train Augustus v2.5.5"

3- were all 15,313 final gene models manually curated in Apollo, or only some?
All models were manually curated.

4 - what criteria were employed for extending 3' UTRs?
The revised manuscript includes a fuller description of the criteria used for manual curation. Please see above.

Reviewer #2: The study of songbirds has contributed critically to our understanding of the neural basis for learning, skilled motor behavior, sexual differentiation of the brain, and countless other topics. The recent availability of multiple high-quality avian genome assemblies has provided a starting point to explore complex questions about the genetic basis and evolution of behavior. The authors provide a high-quality genome assembly for the Bengalese finch, including a set of curated gene annotations and transcriptome data from multiple tissues. This provides a much-needed resource to the many researchers interested in this important model organism. I strongly recommend the paper for submission, following some minor revisions, which I have listed below by line number.

91 - 93 - Please split this run-on sentence into two separate sentences.
Thank you for identifying this error. We have corrected it.

112 - "high coverage," should be high-coverage.
We have made this change in the revised manuscript.

120 - "low coverage," should be low-coverage.
We have made this change in the revised manuscript.

197 - Here the authors state that they manually curated models to ensure completeness and to refine UTR positions. Please provide some brief description as to the logic used to guide the curatorial process. Did the authors use aligned Cufflinks RNA-seq transcripts to guide curation, or aligned models from other species, both, neither, or something else? If it is possible to determine at this point, approximately how many gene models required manual curation?
All genes were curated. The revised manuscript has been modified to indicate this. The revised manuscript also includes a fuller description of the criteria used for manual curation. Please see above.

199 - The authors state that BLASTP was used to align ORFs to the Uniprot-SwissProt protein database. Please specify the parameters used for the BLAST alignment, default or otherwise.
This is now described in the revised manuscript.

Lines 211-212: “...using BLASTP [38] (default parameters except -max_target_seqs 1)”

208 - Here the authors reference Figure 3, which provides a comparison of the Bengalese finch assembly and annotation with the assemblies from the Avian Phylogenomics Project. This figure was helpful for me to get a sense of how this assembly stacks up against previously available avian genomes. I think it would benefit the reader for the authors to provide in text some

qualitative summary of the figure.
A description of how this assembly compares with those in the Avian Phylogenomics project is now provided at the end of ‘Data Description’.

Lines 109-111: “This assembly has coverage and scaffolding length that are on the upper ends of the distribution of assemblies in the Avian Phylogenomics project [26] and has a comparable number of gene models (Fig. 2).”

Table 1:
- Please specify the units for the Age column (e.g. post-hatch days).
- RNA libraries: the table should be alphabetized by tissue type.
- RNA libraries: one of the rows is labeled "Midbrain/brain" where it should be labeled "Midbrain/brainstem."
- RNA libraries: the NCBI records for the RNA-seq samples indicate that the muscle sample is specifically breast muscle. There's no reason not to specify "Breast muscle" in this table also.
We have made these changes to Table 1 in the revised manuscript.

Reviewer #3: Comments to manuscript GIGA-D-17-00224.

General:
The manuscript by Colquitt et al. is a short technical note presenting the development of a draft genome assembly and a set of RNAseq data sets for the Bengalese finch. Besides the assembly stats comparison to previously published avian genomes in the frame-work of the avian genomics consortium, the manuscript contains no analyses. Hence, this review only considers the rationale behind selecting this particular species and the technical aspects of generating the data. The manuscript is well written and easy to follow.

The authors state that the Bangalese finch could develop into a model system for understanding the genetic basis of vocal learning, in particular of song variability and plasticity. In the introduction, the authors make a thorough review of the status of the Bengalese finch in the field of vocal learning. Admittedly, I was initially a bit surprised that the manuscript contained such a detailed introduction describing the study organism but after several rounds of reading I conclude that this strengthens the paper and makes the rationale behind developing genomic tools for this species well supported.

The methods section clearly states how the data was collected and edited before submission to data bases. I could access the data via the links provided so it should be accessible for researchers interested in using these data for analyses. The data was curated with standard methods in the field. My only concerns regard, i) the manual curation of the gene models which could be described in more detail (did you omit ORF:s out of frame?, change the sequence to get ORF in frame?, how was UTR positions determined?, etc.), and, ii) a better description of the rationale behind selecting the particular tissues/sexes for RNAseq.

Detailed comments:
L71 omit 'the'
Thank you for identifying this error. We have corrected it.

L83 Is this statement correct? Several songbirds have very high-quality assemblies available (eg. Ficedula albicollis, Corvus corone).
The reviewer is correct that this statement is no longer accurate. By scaffold N50, the canary (Serinus canaria) and hooded crow (Corvus cornix cornix) both have better assembled genomes. The sentence no longer has this assertion and now reads:

Lines 82-84: "The zebra finch (Taenopygia guttata), another commonly used model for vocal learning, shared a most recent common ancestor with the white-rumped munia ~9 MYA."

L100 It is stated that the Bengalese finch has high level of genetic polymorphism - in the range of outbred human populations. Most songbirds have considerably higher polymorphism levels than that. I would suggest to present this in a different way and give the estimated theta values.

As is suggested by the reviewer, to avoid confusion on this point, a significant elaboration would be required to clearly discuss the details of our measure of nucleotide diversity. Such an elaboration would be beyond the scope of this document and so, we have removed the statement about genetic diversity.

L130 Maybe provide approved animal use protocol (ID number).
Line 128: We have made this change in the revised manuscript.

L182 Ref for TrimGalore!
Line 168: We have made this change in the revised manuscript.

L190-191. Please, provide dates for accession/download since these data bases sometimes change.
We have made this change in the revised manuscript.

Line 194: "Zebra finch EST collection (taeGut2) downloaded from UCSC (on Jan 11, 2015)."

L202-203. As far as I am aware CEGMA is not recommended anymore after the BUSCO tool was developed. The CEGMA part can hence be omitted. Unclear here also how 65% can be complete and 94% partial CEGs?
We have removed the CEGMA analysis.

L332 typos; of, shown?
Line 323: Thank you for identifying this error. We have corrected it.
