# Supplementary material for: Draft genome assembly of the Bengalese finch, Lonchura striata domestica, a model for motor skill variability and learning
Source: Gigascience. 2018 Feb 15;7(3):giy008. doi: 10.1093/gigascience/giy008 (PMC5861438; doi:10.1093/gigascience/giy008)
Supplement: Reviewer_2_Report_(Original_Submission) -- Morgan Wirthlin — 23 Nov 2017 Reviewed [file giy008_reviewer_2_report_(original_submission).pdf]

Reviewer Report

Title: Draft genome assembly of the Bengalese finch, Lonchura striata domestica, a model for motor skill variability and learning

Version: Original Submission **Date:** 23 Nov 2017

Reviewer name: Morgan Wirthlin

Reviewer Comments to Author:

The study of songbirds has contributed critically to our understanding of the neural basis for learning, skilled motor behavior, sexual differentiation of the brain, and countless other topics. The recent availability of multiple high-quality avian genome assemblies has provided a starting point to explore complex questions about the genetic basis and evolution of behavior. The authors provide a high-quality genome assembly for the Bengalese finch, including a set of curated gene annotations and transcriptome data from multiple tissues. This provides a much-needed resource to the many researchers interested in this important model organism. I strongly recommend the paper for submission, following some minor revisions, which I have listed below by line number.

91 - 93 - Please split this run-on sentence into two separate sentences.

112 - "high coverage," should be high-coverage.

120 - "low coverage," should be low-coverage.

197 - Here the authors state that they manually curated models to ensure completeness and to refine UTR positions. Please provide some brief description as to the logic used to guide the curatorial process. Did the authors use aligned Cufflinks RNA-seq transcripts to guide curation, or aligned models from other species, both, neither, or something else? If it is possible to determine at this point, approximately how many gene models required manual curation?

199 - The authors state that BLASTP was used to align ORFs to the Uniprot-SwissProt protein database. Please specify the parameters used for the BLAST alignment, default or otherwise.

208 - Here the authors reference Figure 3, which provides a comparison of the Bengalese finch assembly and annotation with the assemblies from the Avian Phylogenomics Project. This figure was helpful for me to get a sense of how this assembly stacks up against previously available avian genomes. I think it would benefit the reader for the authors to provide in text some qualitative summary of the figure.

Table 1:
- Please specify the units for the Age column (e.g. post-hatch days).
- RNA libraries: the table should be alphabetized by tissue type.

- RNA libraries: one of the rows is labeled "Midbrain/brain" where it should be labeled "Midbrain/brainstem."
- RNA libraries: the NCBI records for the RNA-seq samples indicate that the muscle sample is specifically breast muscle. There's no reason not to specify "Breast muscle" in this table also.

Level of Interest

Please indicate how interesting you found the manuscript: An article of importance in its field

Quality of Written English

Please indicate the quality of language in the manuscript: Acceptable

Declaration of Competing Interests

Please complete a declaration of competing interests, considering the following questions:

- Have you in the past five years received reimbursements, fees, funding, or salary from an organisation that may in any way gain or lose financially from the publication of this manuscript, either now or in the future?
- Do you hold any stocks or shares in an organisation that may in any way gain or lose financially from the publication of this manuscript, either now or in the future?
- Do you hold or are you currently applying for any patents relating to the content of the manuscript?
- Have you received reimbursements, fees, funding, or salary from an organization that holds or has applied for patents relating to the content of the manuscript?
- Do you have any other financial competing interests?
- Do you have any non-financial competing interests in relation to this paper?

If you can answer no to all of the above, write 'I declare that I have no competing interests' below. If your reply is yes to any, please give details below.

I declare that I have no competing interests.

I agree to the open peer review policy of the journal. I understand that my name will be included on my report to the authors and, if the manuscript is accepted for publication, my named report including any attachments I upload will be posted on the website along with the authors' responses. I agree for my report to be made available under an Open Access Creative Commons CC-BY license (http://creativecommons.org/licenses/by/4.0/). I understand that any comments which I do not wish to

be included in my named report can be included as confidential comments to the editors, which will not be published.

I agree to the open peer review policy of the journal

To further support our reviewers, we have joined with Publons, where you can gain additional credit to further highlight your hard work (see: https://publons.com/journal/530/gigascience). On publication of this paper, your review will be automatically added to Publons, you can then choose whether or not to claim your Publons credit. I understand this statement.

Yes
