# Supplementary material for: Draft genome assembly of the Bengalese finch, Lonchura striata domestica, a model for motor skill variability and learning
Source: Gigascience. 2018 Feb 15;7(3):giy008. doi: 10.1093/gigascience/giy008 (PMC5861438; doi:10.1093/gigascience/giy008)
Supplement: Reviewer_3_Report_(Original_Submission) -- Niclas Backström — 26 Nov 2017 Reviewed [file giy008_reviewer_3_report_(original_submission).pdf]

Reviewer Report

Title: Draft genome assembly of the Bengalese finch, Lonchura striata domestica, a model for motor skill variability and learning

Version: Original Submission **Date:** 26 Nov 2017

Reviewer name: Niclas Backström

Reviewer Comments to Author:

Comments to manuscript GIGA-D-17-00224.

General:
The manuscript by Colquitt et al. is a short technical note presenting the development of a draft genome assembly and a set of RNAseq data sets for the Bengalese finch. Besides the assembly stats comparison to previously published avian genomes in the frame-work of the avian genomics consortium, the manuscript contains no analyses. Hence, this review only considers the rationale behind selecting this particular species and the technical aspects of generating the data. The manuscript is well written and easy to follow.

The authors state that the Bangalese finch could develop into a model system for understanding the genetic basis of vocal learning, in particular of song variability and plasticity. In the introduction, the authors make a thorough review of the status of the Bengalese finch in the field of vocal learning. Admittedly, I was initially a bit surprised that the manuscript contained such a detailed introduction describing the study organism but after several rounds of reading I conclude that this strengthens the paper and makes the rationale behind developing genomic tools for this species well supported.

The methods section clearly states how the data was collected and edited before submission to data bases. I could access the data via the links provided so it should be accessible for researchers interested in using these data for analyses. The data was curated with standard methods in the field. My only concerns regard, i) the manual curation of the gene models which could be described in more detail (did you omit ORF:s out of frame?, change the sequence to get ORF in frame?, how was UTR positions determined?, etc.), and, ii) a better description of the rationale behind selecting the particular tissues/sexes for RNAseq.

Detailed comments:
L71 omit 'the'

L83 Is this statement correct? Several songbirds have very high-quality assemblies available (eg. Ficedula albicollis, Corvus corone).

L100 It is stated that the Bengalese finch has high level of genetic polymorphism - in the range of

outbred human populations. Most songbirds have considerably higher polymorphism levels than that. I would suggest to present this in a different way and give the estimated theta values.

L130 Maybe provide approved animal use protocol (ID number).

L182 Ref for TrimGalore!

L190-191. Please, provide dates for accession/download since these data bases sometimes change.

L202-203. As far as I am aware CEGMA is not recommended anymore after the BUSCO tool was developed. The CEGMA part can hence be omitted. Unclear here also how 65% can be complete and 94% partial CEGs?

L332 typos; of, shown?

Level of Interest

Please indicate how interesting you found the manuscript: An article of limited interest

Quality of Written English

Please indicate the quality of language in the manuscript: Acceptable

Declaration of Competing Interests

Please complete a declaration of competing interests, considering the following questions:

- Have you in the past five years received reimbursements, fees, funding, or salary from an organisation that may in any way gain or lose financially from the publication of this manuscript, either now or in the future?
- Do you hold any stocks or shares in an organisation that may in any way gain or lose financially from the publication of this manuscript, either now or in the future?
- Do you hold or are you currently applying for any patents relating to the content of the manuscript?
- Have you received reimbursements, fees, funding, or salary from an organization that holds or has applied for patents relating to the content of the manuscript?
- Do you have any other financial competing interests?
- Do you have any non-financial competing interests in relation to this paper?

If you can answer no to all of the above, write 'I declare that I have no competing interests' below. If your reply is yes to any, please give details below.

I declare that I have no competing interests

I agree to the open peer review policy of the journal. I understand that my name will be included on my report to the authors and, if the manuscript is accepted for publication, my named report including any attachments I upload will be posted on the website along with the authors' responses. I agree for my report to be made available under an Open Access Creative Commons CC-BY license (http://creativecommons.org/licenses/by/4.0/). I understand that any comments which I do not wish to be included in my named report can be included as confidential comments to the editors, which will not be published.

I agree to the open peer review policy of the journal

To further support our reviewers, we have joined with Publons, where you can gain additional credit to further highlight your hard work (see: https://publons.com/journal/530/gigascience). On publication of this paper, your review will be automatically added to Publons, you can then choose whether or not to claim your Publons credit. I understand this statement.

Yes
